# NSUN2‐mediated m^5^C RNA methylation dictates retinoblastoma progression through promoting PFAS mRNA stability and expression

**DOI:** 10.1002/ctm2.1273

**Published:** 2023-05-25

**Authors:** Sipeng Zuo, Lin Li, Xuyang Wen, Xiang Gu, Ai Zhuang, Rui Li, Fuxiang Ye, Shengfang Ge, Xianqun Fan, Jiayan Fan, Peiwei Chai, Linna Lu

**Affiliations:** ^1^ Department of Ophthalmology Ninth People's Hospital Shanghai Jiao Tong University School of Medicine Shanghai People's Republic of China; ^2^ Shanghai Key Laboratory of Orbital Diseases and Ocular Oncology Shanghai People's Republic of China

**Keywords:** m^5^C modification, metabolic reprogramming, NSUN2, PFAS

## Abstract

**Background:**

The precise temporal and spatial regulation of N^5^‐methylcytosine (m^5^C) RNA modification plays essential roles in RNA metabolism, and is necessary for the maintenance of epigenome homeostasis. Howbeit, the mechanism underlying the m^5^C modification in carcinogenesis remains to be fully addressed.

**Methods:**

Global and mRNA m^5^C levels were determined by mRNA isolation and anti‐m^5^C dot blot in both retinoblastoma (RB) cells and clinical samples. Orthotopic intraocular xenografts were established to examine the oncogenic behaviours of RB. Genome‐wide multiomics analyses were performed to identify the functional target of NSUN2, including proteomic analysis, transcriptome screening and m^5^C‐methylated RNA immunoprecipitation sequencing (m^5^C‐meRIP‐seq). Organoid‐based single‐cell analysis and gene‐correlation analysis were performed to verify the NSUN2/ALYREF/m^5^C‐PFAS oncogenic cascade.

**Results:**

Herein, we report that NSUN2‐mediated m^5^C RNA methylation fuels purine biosynthesis during the oncogenic progression of RB. First, we discovered that global and mRNA m^5^C levels were significantly enriched in RBs compared to normal retinas. In addition, tumour‐specific NSUN2 expression was noted in RB samples and cell lines. Therapeutically, targeted correction of NSUN2 exhibited efficient therapeutic efficacy in RB both in vitro and in vivo. Through multiomics analyses, we subsequently identified *phosphoribosylformylglycinamidine synthase* (PFAS), a vital enzyme in purine biosynthesis, as a downstream candidate target of NSUN2. The reintroduction of PFAS largely reversed the inhibitory phenotypes in NSUN2‐deficient RB cells, indicating that PFAS was a functional downstream target of NSUN2. Mechanistically, we found that the m^5^C reader protein ALYREF was responsible for the recognition of the m^5^C modification of PFAS, increasing its expression by enhancing its RNA stability.

**Conclusions:**

Conclusively, we initially demonstrated that NSUN2 is necessary for oncogenic gene activation in RB, expanding the current understanding of dynamic m^5^C function during tumour progression. As the NSUN2/ALYREF/m^5^C‐PFAS oncogenic cascade is an important RB trigger, our study suggests that a targeted m^5^C reprogramming therapeutic strategy may be a novel and efficient anti‐tumour therapy approach.

## Graphical abstract

- NSUN2‐mediated m5C modification accelerates retinoblastoma progression.
- NSUN2 is necessary for PFAS activation in vitro and in vivo.
- m5C modification is important for purine synthesis in retinoblastoma.

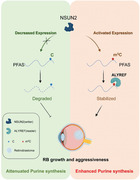

## 1 INTRODUCTION

N5‐methylcytosine (m^5^C) is an important posttranscriptional RNA modification pattern identified in tRNAs, rRNAs and mRNAs.^1^, ^2^, ^3^, ^4^ The catalysis of m^5^C modification is governed by enzymes in the NOL1/NOP2/SUN domain (NSUN) family and DNA methyltransferase DNMT2.^1^, ^5^, ^6^ Notably, ALYREF and YBX1 have been revealed to play important roles in the recognition of m^5^C‐methylated transcripts (also termed ‘readers’) and subsequently regulate their expression by controlling their RNA stability.^7^, ^8^ Recently, m^5^C has been revealed to play an essential role in RNA metabolism, including mRNA export and posttranscriptional regulation.^1^, ^9^, ^10^ m^5^C RNA modification has also been revealed as an important physiological regulator in HIV infection, neuronal synaptic signalling, vascular endothelial inflammation and T cell activation, which further underscores the importance of m^5^C modification in the maintenance of intracellular homeostasis.^11^, ^12^, ^13^, ^14^ Importantly, RNA epitranscriptomic modification is in a dynamic and reversible state, participating in the process of gene expression through intervening in RNA metabolism (e.g. RNA maturation, editing, localization, stability and translation).^15^ As the most plentiful epitranscriptomic modification on mRNAs, m^6^A has been extensively studied in gene expression in cancer.^16^ Notably, as another important form of RNA modification, the regulatory roles of m^5^C remain to be fully addressed.

Importantly, NSUN2‐mediated m^5^C modification is involved in cell proliferation and invasion in a variety of tumours, by activating oncogenes or inhibiting tumour suppressors. For example, NSUN2 induces m^5^C modification of growth factor receptor‐bound protein 2 (GRB2) and stabilizes its mRNA, facilitating the tumorigenesis of oesophageal squamous cell carcinoma (ESCC).^17^ In addition, NSUN2 promotes the expression of heparin‐binding growth factor (HDGF) and promotes pathogenesis of the bladder cancer.^18^ Moreover, NSUN2 promotes cell migration by methylating autotaxin mRNA in human glioma cells.^19^ Therefore, these results indicate that aberrant m^5^C modification functions as an important malignant switch during oncogenesis.

Retinoblastoma (RB) is the most frequent intraocular malignancy in childhood, causing blindness and even death.^20^, ^21^ To date, loss of RB1 (> 90%) and MYCN amplification (∼10%) have been considered the oncogenic driving events, leading to enhanced cell cycle turnover and activation of oncogenes.^22^, ^23^ Notably, recent studies have shown that epigenetic deficiency also participates in RB tumour progression.^24^, ^25^, ^26^ For example, downregulated DNA methylation of spleen tyrosine kinase leads to its overexpression, contributing to its activation of cell cycle progression.^27^ Moreover, an abnormal chromosomal state of the chr12p13.32 locus gives rise to the epigenetic activation of polypeptide N‐acetylgalactosaminyltransferase 8 (GALNT8), which supports the malignant growth of RB.^28^ More recently, lncCANT1 was found to be downregulated in RB, which is associated with activation of PI3K signalling.^29^ Taken together, these data indicate that epigenetic abnormalities are also involved in oncogenic transformation in RB. However, the role of m^5^C modification on RNA outside of the epigenetic context remains enigmatic.

Thus, we aimed to identify the oncogenic function of m^5^C modification in RB. In this study, we found that enhanced NSUN2 leads to an elevated level of m^5^C modification, which supported cell growth both in vitro and in vivo. Using genome‐wide proteomic analysis, transcriptome screening, m^5^C‐meRIP‐seq and organoid‐based single‐cell analysis, we found that NSUN2 enhances the expression of phosphoribosylformylglycinamidine synthase (PFAS), which is a vital enzyme in purine biosynthesis, serving as a functional downstream target of NSUN2. Mechanistically, we found that the m^5^C reader protein ALYREF was responsible for the recognition of the m^5^C modification of PFAS, increasing its expression by enhancing its RNA stability. Overall, we revealed that m^5^C modification on mRNA is important for purine synthesis, which bridges the current understanding of RNA modification and metabolic reprogramming. Since the NSUN2/ALYREF/m^5^C‐PFAS oncogenic cascade is an important trigger of RB, our study provides a novel targeted m^5^C reprogramming therapeutic strategy, which may potentially be an efficient anti‐tumour therapy approach.

## 2 MATERIALS AND METHODS

### 2.1 Cell lines

The RB cell line Y79 was acquired from American Type Culture Collection. The RB cell line WERI‐Rb1 and the adult retinal pigment epithelium cell line ARPE‐19 were purchased from Cell Bank (Chinese Academy of Sciences, Shanghai, China). The cells were cultured in RPMI 1640 medium (Invitrogen, Carlsbad, USA) supplemented with 10% foetal bovine serum (FBS; Gibco, USA) and 1% penicillin/streptomycin. The cultures were cultured in a humidified atmosphere at 37°C with 5% CO_2_. The cells were authenticated by STR profiling.

### 2.2 Tissue collection

RB tumour tissues and adjacent normal retinas were collected in the Department of Ophthalmology, Ninth People's Hospital, Shanghai Jiao Tong University School of Medicine. We detached the tissues from the vitreous cavity of the eyeball. After snap‐frozen in moderate liquid nitrogen, fresh tissue samples were placed at −80°C for long‐term storage. Prior to surgery, patient consent documents from the Institutional Research Ethics Committee were obtained. The clinicopathological information for these tissue specimens is described in Table S1.

### 2.3 Immunofluorescence

Immunofluorescence was performed as described previously.^30^ Fixed RB tissues were embedded using embedding medium (Sakura, Japan). The specimens were cut into 2‐μm‐thick sections. Then, the tissues were fixed on the polylysine‐coated slides at 60°C for 30 min. The slides were washed using PBS for 10 min three times. The sections were blocked with normal goat serum (Gibco) at room temperature (RT) for 1 h. After washed with PBS three times, the samples were incubated with anti‐NSUN2(20854, Proteintech, China) or anti‐PFAS (76957, CST, USA) antibody overnight at 4°C. After being washed with PBS three times, the tissues were incubated with secondary antibodies for 1.5 h at RT. Eventually, image acquisition was performed using a fluorescence microscope.

### 2.4 Dot blot assay

Total RNA was extracted using TRIzol reagent (Invitrogen) according to the standard manufacturer's protocol and then quantified and diluted in 10 mM Tris‐EDTA buffer. The indicated amounts of RNA samples were loaded onto Hybond‐N + membranes (FFN10, Beyotime, China). The membrane was crosslinked at 254 nm UV for 60 s after a short drying process, blocked with 5% milk for 1.5 h at RT and incubated with an anti‐m^5^C antibody (ab214727, Abcam, USA) at 4°C overnight. After three washes with TBST (Thermo Fisher Scientific, USA), the membranes were incubated with HRP‐conjugated anti‐rabbit IgG (SA00001‐2, Proteintech) for 1.5 h at RT and then visualized using an enhanced chemiluminescence kit (WBKLS0100, Thermo Fisher Scientific) and a detection instrument (Tanon Science, Shanghai, China).

### 2.5 Western blotting

Western blotting was performed as described previously.^31^ Protein was obtained at the indicated times using lysis buffer containing proteinase inhibitors (87785, Thermo Fisher Scientific). The obtained supernatant was separated via 10% SDS‐PAGE gels (Takara, Japan) and transferred to PVDF membranes (Millipore, USA). Following blocking by 10% milk for 40 min at RT, the proteins were incubated overnight at 4°C with anti‐NSUN2(20854, Proteintech), anti‐PFAS (76957, CST), anti‐GAPDH (60004‐1‐Ig, Proteintech), anti‐β‐actin (66009‐1‐Ig, Proteintech) and anti‐ALYREF (ab202894, Abcam). After incubated with HRP‐conjugated anti‐rabbit IgG (SA00001‐2, Proteintech) or HRP‐conjugated anti‐mouse IgG (SA00001‐1, Proteintech) at RT for 1.5 h. Subsequently, signal detection was performed using an ECL kit and visualized with the detection instrument.

### 2.6 RNA extraction and qRT‐PCR

Total RNA was extracted using TRIzol reagent as above described. cDNA was then synthesized using a PrimeScript RT Reagent Kit (Takara). qRT‒PCR was executed on a Roche LightCycler 480 system (ABI, USA). The primers used in this study are listed in Table S2.

#### 2.6.1 mRNA isolation

According to the manufacturer guideline and previous studies,^32^ the mRNA isolation kit (12603ES24, Yeasen, China) was used. The 50 μL mRNA capture beads were mixed completely with 4 μg/50 μL total RNA. Then, the mixture was heated up to 65°C for 5 min. After short cooling at RT, the beads were washed twice with 200 μL wash buffer. The 50 μL tris buffer was added into the beads and heated up to 80°C for 2 min to elute mRNA. Then, the 50 μL beads binding buffer was added into the mRNA and incubated at RT for 5 min. Then, the washing was repeated once again. At last, the beads were incubated at 80°C for 2 min to separate mRNA.

#### 2.6.2 Cytoplasmic and Nuclear RNA Purification

According to the manufacturer guideline, the Cytoplasmic and Nuclear RNA Purification Kit (21000, Norgen, USA) was used. Briefly, the culture was dissolved with 200 μL lysis buffer. After a short vortex and centrifugation, the cytoplasmic RNA was transferred into another tube. A moderate buffer solution was added into the cytoplasmic and nuclear RNA. The mixture was centrifuged for 1 min at 3500 g. Then, column wash and RNA elution were performed again to separate cytoplasmic and nuclear RNA. The RNA was placed at −80°C for long‐term storage.

#### 2.6.3 Inosinemonphosphate detection

According to the published method,^33^ approximately 10^7^ cells were harvested and treated with 500 μL .4 mol/L perchloric acid solution. After ultrasonic treatment, the protein concentration was detected through a BCA assay. Hundred microliters supernate was mixed with 100 μL .4 mol/L potassium hydroxide solution, centrifuged for 10 min at 12 000 rpm at 4°C. The supernate was analysed using waters 2695 high‐performance liquid chromatograph (Waters Alliance, USA). After normalization by protein concentration, inosinemonphosphate (IMP) concentration was analysed and calculated.

### 2.7 Lentivirus packaging and stable cell lines

A combination of Lipofectamine 3000 reagent (Invitrogen) and Opti‐MEM I Reduced Serum Medium (Gibco) was employed to transfect 293 cells with 3 μg of plasmid and 6 μg of PsPax plasmid and 3 μg of pMD2.D plasmid. After 4 h, the medium was replaced with 12 mL of fresh medium. The medium was gathered at 72 h. Prior to transduction, the cell medium was replaced with a virus‐containing medium containing 3 ng/mL polybrene (Sigma‒Aldrich, USA). The medium was refreshed after transduction. The selection was performed with 3 μg/mL puromycin for 2 weeks and 1 μg/mL puromycin (InvivoGen, USA) for a long time.

### 2.8 siRNA interference

ALYREF siRNA was purchased from Genomeditech (Shanghai, China). The sequences of the selected siRNAs are listed in Table S2. Lipofectamine 3000 reagent was used to transfect cells with siRNA according to the manufacturer's instructions.

### 2.9 Cell proliferation assay

In total, 3000−5000 RB cells were seeded into per well of 96‐well plates. Then, 10 μL of Cell Counting Kit‐8 (CCK8, Dojindo, Japan) reagent was added to each well at the indicated time. After incubation for 3 h, the absorbance of 450 nm was detected with a microplate reader (ELX800, BioTek, USA).

### 2.10 Soft agar assay

A soft agar assay was performed as described previously.^34^ Briefly, the mixture of 1 mL of .8% agar (Sigma‒Aldrich) and 1 mL complete medium was spread into a 6‐well plate. After 10 min at RT, 1.5 × 10^4^ RB cells were mixed .75 mL complete medium and .75 mL .4% agar and seeded. Every 3 days, 150 μL of complete medium was added to keep the soft agar wet. The 6‐well plates were cultured for a month. After that, the soft agar was washed three times and then stained with a .1% crystal violet solution for 3 h. Images were captured, and the number of colonies was counted using ImageJ software (7.0).

### 2.11 Xenograft models and immunohistochemistry staining

Animal ethics were approved by the Institutional Animal Care and Use Committee of the Ninth People's Hospital, Shanghai Jiao Tong University School of Medicine. Eighteen 5‐week‐old BALB/c nude mice were obtained from the Model Animal Research Center of Shanghai Jiao Tong University School of Medicine. After a week of environmental adaptation, approximately 5−10 × 10^4^ Y79 cells from each group were injected into the eyeball with a sharp 30‐gauge injection needle. The infected eyes were protected with ophthalmic bacitracin ointment. Four weeks after tumour cell injection, the nude mice were sacrificed, and the eyeballs were excised, weighed and photographed. The tumours were embedded in paraffin and then sliced into 2 μm thick sections. Next, the slides were incubated with the specific primary antibody at 4°C overnight, then incubated with biotinylated goat anti‐rabbit serum streptavidin–peroxidase conjugate at RT for .5 h. Finally, after sections were developed with diaminobenzidine and counterstained with haematoxylin, images were captured.

### 2.12 RNA sequencing

Briefly, total RNA was isolated from the cultured cells using a TRIzol reagent (Sigma‒Aldrich). Then, we confirmed the RNA integrity using a 2100 Bioanalyzer (Agilent Technologies, USA) and detected the RNA concentration using a Qubit 2.0 fluorometer with a Qubit RNA assay kit (Q32855, Invitrogen). Then, sequencing libraries were generated using an Illumina TruSeq RNA Sample Prep Kit (RS‐122‐2001, Illumina, USA). Finally, the libraries were sequenced on the Illumina HiSeq 3000 platform (Illumina).

### 2.13 Isobaric tags for relative and absolute quantitation proteomic analysis

The control and NSUN2‐deficient Y79 cells were lysed with SDT buffer (4% SDS, 100 mM Tris‐HCl, 1 mM DTT, pH 7.6). Protein supernatants were subjected to protein digestion, tandem mass tag labelling, fractionation, LC‒MS/MS analysis, protein identification and protein quantitation.

### 2.14 MeRIP‐seq and data analysis

In brief, RNA was randomly fragmented to approximately 200 nt using RNA Fragmentation Reagents, and m^5^C antibody was conjugated to protein A/G beads at RT for 1 h. The RNA fragments were incubated with the beads at 4°C overnight. Then, the captured RNA was eluted from the beads and extracted with TRIzol reagent. Both the input sample and the m^5^C IP sample were subjected to library generation. Libraries were qualified using an Agilent 2100 bioanalyzer (Agilent, USA) and sequenced on a NovaSeq 6000 platform (Illumina). After quality control and removal of low‐quality reads, the clean reads of all libraries were aligned to the reference genome (HG19) using HISAT2 software (v2.0.4) 36. Methylated sites on RNAs (peaks) were identified and differentially methylated sites were identified using diffReps. Moreover, GO and pathway enrichment analyses were performed using the differentially methylated protein‐coding genes.

### 2.15 RNA‐binding protein immunoprecipitation‐qPCR

A Magana RIP Quad kit (17−704, Millipore) was used to examine RNA‐binding proteins on specific genes according to the manufacturer's protocol. Briefly, a total of 3.0 × 10^7^ cells were lysed to obtain 250 μL of RIP lysate, of which 20 μL was kept as an input control and 200 μL was enriched with antibody‐ or rabbit IgG‐conjugated Protein A/G Magnetic Beads in IP buffer supplemented with RNase inhibitor at 4°C overnight. The immunoprecipitated RNA was digested and purified with the beads. The purified RNA and the input control were further analysed via qPCR. The following primary antibodies were used in the study: anti‐ALYREF (ab202894, Abcam), anti‐YBX1 (ab76149, Abcam) and anti‐MTR4 (ab70551, Abcam).

### 2.16 Enzyme‐linked immunosorbent assay

According to the manufacturer's protocol (HBP31539R, HBP36959R, Huabang Bio, China), a standard curve was generated. Then, sample solutions were prepared through multigelation and added to the detection plates. The reaction system was incubated at 37°C for 1 h with 100 μL HRP conjugated antibody in each well. After five washes with wash buffer, 100 μL of chromogen solution was added to each well. The mixture was gently mixed and incubated for 15 min at 37°C. Finally, within 15 min, the absorbance of each well was measured at 450 nm using a microtiter plate reader.

### 2.17 RNA stability assay

A total of 500−1000 Y79 cells were seeded into 6‐well plates for 24 h. Cells were treated with 5 μg/mL actinomycin D (Sigma‒Aldrich) and collected at the indicated time points. RNA isolation and qRT‒PCR were performed further as described above.

### 2.18 Statistical analyses

Prism 7.0 software (GraphPad, USA) was used for statistical analyses. Quantification data are presented as the mean ± SD, and differences between the two groups were compared with an unpaired Student's *t*‐test. Differences were considered significant at *p* value < .05, and asterisks denote statistical significance (*p < .05, **p < .01, ***p < .001 and ****p < .0001).

## 3 RESULTS

### 3.1 Increased NSUN2 expression enhances the m5C modification level in RB

**FIGURE 1:**
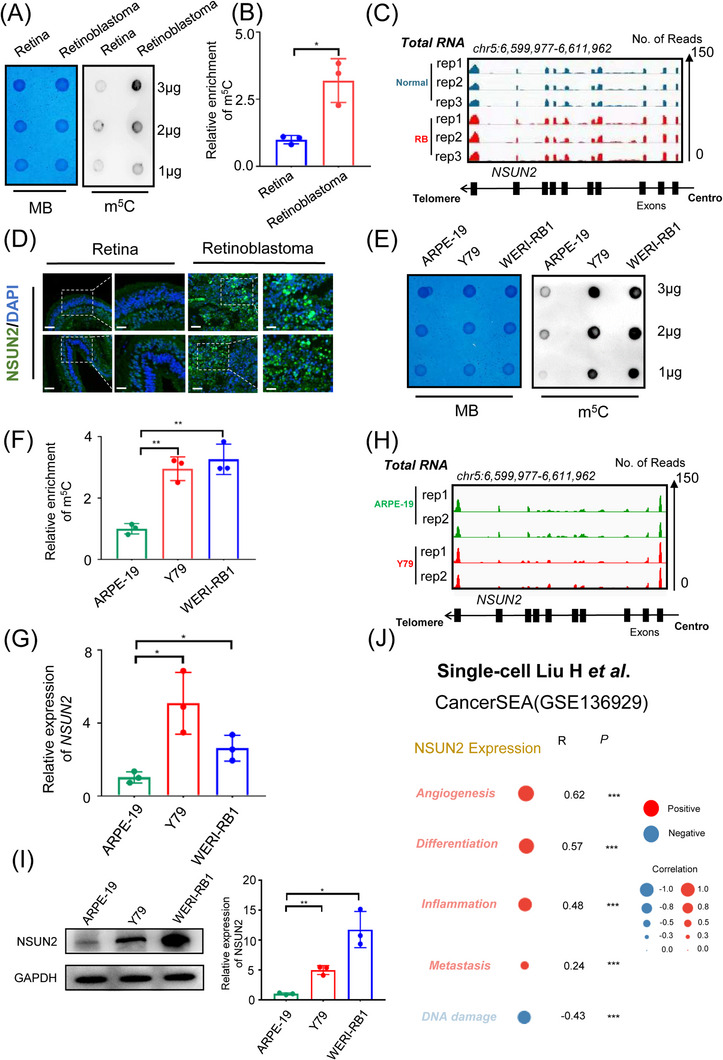
Increased NSUN2 expression enhances the m^5^C modification level in retinoblastoma (A and B). Dot blot showing the m^5^C signal compared to the methylene blue signal in retinoblastoma and normal retina tissue. The data are presented as the mean ± SD of experimental triplicates. Significance was determined by an unpaired two‐tailed Student's *t* test. *p < .05. (C) Integrative Genomics Viewer (IGV) showing higher expression of NSUN2 in retinoblastoma tissue than in normal retinal tissue. (D) Immunofluorescence of NSUN2 (green) and DAPI staining (blue) in tumour and normal samples. Scale bars: left panel, 50 μm; right panel, 25 μm. (E and F) Dot blot showing the m^5^C signal compared to the methylene blue signal in retinoblastoma cell lines and retinal pigment epithelium cell lines. The data are presented as the mean ± SD of experimental triplicates. Significance was determined by an unpaired two‐tailed Student's *t* test. **p < .01. (G) qPCR data showing NSUN2 expression in retinoblastoma cells relative to ARPE‐19 cells. The data are presented as the mean ± SD of experimental triplicates. Significance was determined by an unpaired two‐tailed Student's *t* test. *p < .05. (H) IGV showing higher expression of NSUN2 in retinoblastoma cell lines relative to the retinal pigment epithelium cell line. (I) Western blot data and statistical analysis showing NSUN2 expression in retinoblastoma cells relative to ARPE‐19 cells. The data are representative of experimental triplicates. The data are presented as the mean ± SD of experimental triplicates. Significance was determined by an unpaired two‐tailed Student's *t* test. *p < .05, **p < .01. (J) Single‐cell transcriptome profiling revealing the correlation between relative NSUN2 protein expression and different biological processes in retinoblastoma. ***p < .001.

To explore the potential functions of m^5^C modification during the pathogenesis of RB, we first compared the m^5^C modification levels between RB samples and normal retinas. As demonstrated by anti‐m^5^C dot blot assay results, RB samples presented with elevated global m^5^C levels (Figure 1A, B). Importantly, the methyltransferase NSUN2 also showed increased signal intensity in the clinical RB samples, as demonstrated by RNA‐seq (Figure 1C, deposited in GEO database, GSE111168) and immunofluorescence assays (Figure 1D and Figure S1A). Moreover, RB cell lines (Y79 and WERI‐RB1) presented with increased m^5^C levels, compared with normal retinal cell line (ARPE‐19) (Figure 1E, F). Notably, RNA m^5^C dot blot mainly reflects m^5^C levels of rRNA and tRNA, rather than mRNA m^5^C modification.^35^ To explore the potential functions of m^5^C modification in the mRNA fate regulation during the pathogenesis of RB, we then isolated mRNA and compared the mRNA m^5^C modification levels. As a result, enhanced mRNA m^5^C levels were observed in RB samples (Figure S1B, C) and cell lines (Figure S1D, E). Importantly, a significant elevation in *NSUN2* expression was noted in these RB cell lines at both the RNA (Figure 1G, H, GEO: GSE214685) and protein (Figure 1I) levels.

Interestingly, according to single‐cell analyses in RB organoids,^27^ the overexpression of NSUN2 was correlated with enhanced scores for several oncogenic events (measured in CancerSEA platform,^36^ Figure 1J and Figure S2), including angiogenesis (*R* = .62, *p* < .001), differentiation (*R* = .57, *p* < .001), inflammation (*R* = .48, *p* < .001) and metastasis (*R* = .24, *p* < .001). Conclusively, these observations indicate that NSUN2 overexpression potentially serves as an “oncogenic trigger” in the pathogenesis of RB.

### 3.2 NSUN2 fuels malignant proliferation of RB in vitro and in vivo

**FIGURE 2:**
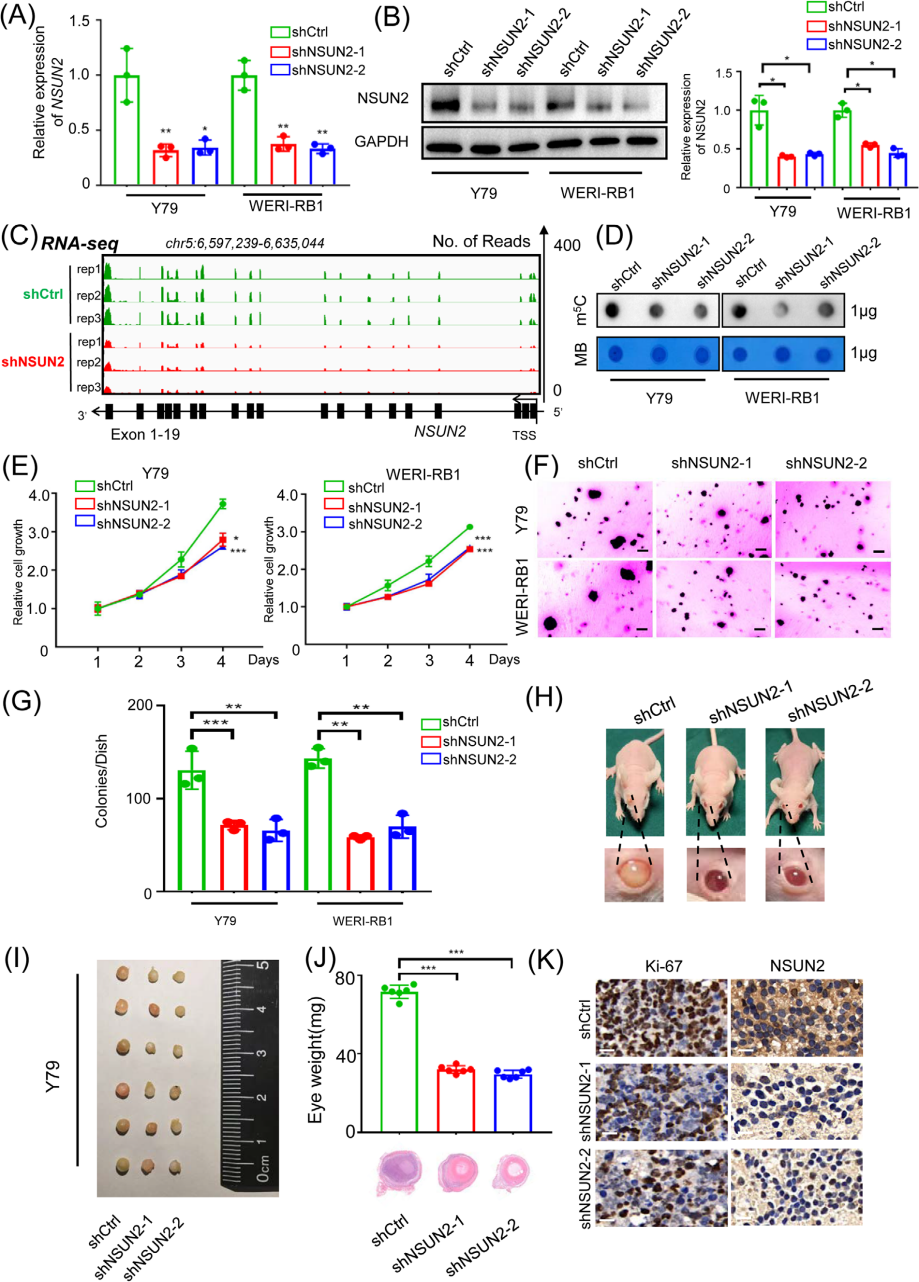
NSUN2 promotes malignant proliferation of retinoblastoma in vitro and in vivo. (A) qPCR data showing NSUN2 expression in retinoblastoma cells (Y79 and WERI‐RB1) following NSUN2 knockdown. The data are presented as the mean ± SD of experimental triplicates. Significance was determined by an unpaired two‐tailed Student's *t* test. *p < .05, **p < .01. (B) Western blot and statistical analysis showing NSUN2 expression in retinoblastoma cells (Y79 and WERI‐RB1) following NSUN2 knockdown. The data are representative of experimental triplicates. The data are presented as the mean ± SD of experimental triplicates. Significance was determined by an unpaired two‐tailed Student's *t* test. *p < .05. (C) IGV showing the expression of NSUN2 in retinoblastoma cells (Y79) following NSUN2 knockdown. (D) Dot blot showing the m^5^C signal relative to the methylene blue signal in retinoblastoma cells (Y79 and WERI‐RB1) following NSUN2 knockdown. (E) A CCK‐8 assay was employed to evaluate the proliferation of retinoblastoma cells (Y79 and WERI‐RB1) after NSUN2 knockdown. The data are presented as the mean ± SD of experimental triplicates. Significance was determined by an unpaired two‐tailed Student's *t* test. *p < .05, ***p < .001. (F and G) A soft agar assay was employed to evaluate the proliferation of retinoblastoma cells (Y79 and WERI‐RB1) following NSUN2 knockdown. Representative images from three experimental replicates are shown. The data are presented as the mean ± SD. Significance was determined by an unpaired two‐tailed Student's *t* test. **p < .01, ***p < .001. (H and I) Images of BALB/c nude mice and eyeballs containing xenografts derived from NSUN2‐deficient Y79 cells. Representative images from six biological replicates are shown. (J) Statistical analysis of the eyeball weight data. H&E staining to evaluate tumour formation. The data are presented as the mean ± SD. Significance was determined by an unpaired two‐tailed Student's *t* test. ***p < .001. (K) Immunohistochemical staining images showing Ki‐67 and NSUN2 expression in the control group and NSUN2 knockdown group. Scale bars: 50 μm.

To explore the role of NSUN2 in the malignant transformation of RB, we first silenced NSUN2 by transfecting cells with two individual shRNAs (Table S2). Importantly, a dramatic decrease in *NSUN2* expression in RB cell lines was noted after transfection with the two shRNAs, as demonstrated by qRT‒PCR (Figure 2A), western blotting (Figure 2B) and RNA‐seq (Figure 2C). Concordantly, the global and mRNA m^5^C level was significantly decreased following the silencing of NSUN2 in two RB cell lines (Figure 2D and Figure S3). Cell growth assays revealed a remarkably decreased proliferation rate compared to the vector group (Figure 2E). Moreover, silencing of NSUN2 significantly inhibited the anchorage‐independent growth of RB cells (Figure 2F, G). These results support the notion that NSUN2 functions as a necessary oncogenic accelerator for the malignant transformation of RB in vitro. To assess their in vivo tumour formation ability, we injected NSUN2‐silenced and control Y79 cells into the eyeballs of nude mice and monitored tumour growth in orthotopic xenograft models (Figure 2H). Notably, smaller and lighter tumours formed within the eyeball in the NSUN2‐deficient group than in the empty vector group (Figure 2I, J). Consistently, the Ki67‐positive rate (Figure 2K, left) and NSUN2 expression (Figure 2K, right) were significantly decreased after NSUN2 inhibition. Conclusively, targeted correction of NSUN2 exhibited efficient therapeutic efficacy in vitro and in vivo.

### 3.3 PFAS modulates the m^5^C modification of NSUN2

**FIGURE 3:**
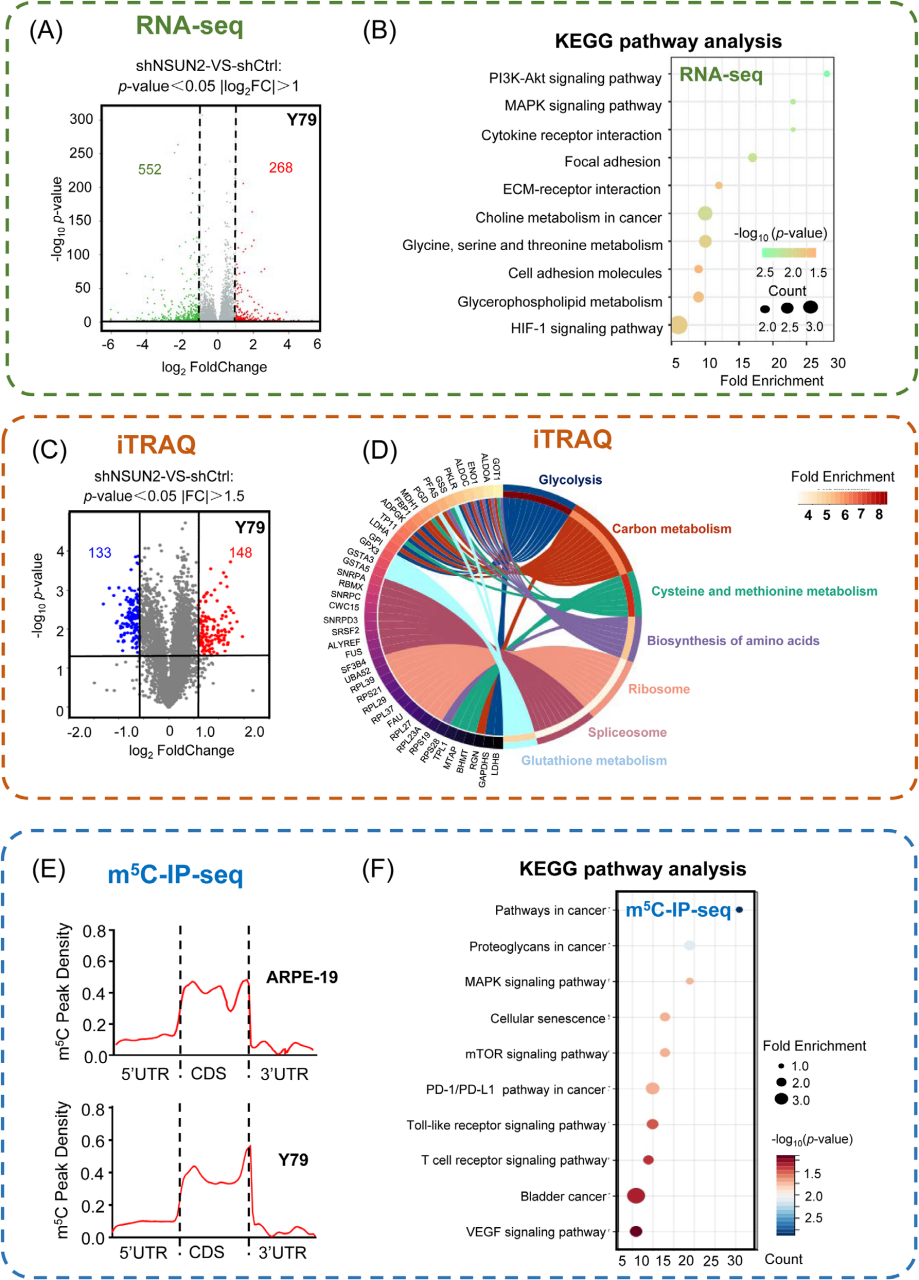
Multiomics analyses were performed to determine the potential RNA targets of NSUN2. (A) Volcano plots showing NSUN2‐regulated genes in NSUN2‐deficient retinoblastoma (Y79) cells. (B) KEGG pathway analysis of NSUN2‐regulated genes in NSUN2‐deficient retinoblastoma (Y79) cells. (C) Volcano plots showing NSUN2‐regulated proteins in NSUN2‐deficient retinoblastoma (Y79) cells. (D) GO analysis of NSUN2‐regulated proteins in NSUN2‐deficient retinoblastoma (Y79) cells. (E) m^5^C‐meRIP‐seq data showing the peak density of m^5^C sites. Biological duplicates were analysed. (F) KEGG pathway analysis of m^5^C‐modified genes in retinoblastoma (Y79) cells and normal retina tissues.

To explore the mechanism by which NSUN2 promotes the pathogenesis of RB, we performed multiomics analyses to determine potential RNA targets of NSUN2, including transcriptome analysis (RNA‐seq; Figure 3A, B, deposited in GSE214685), proteomic analysis (isobaric tags for relative and absolute quantitation [iTRAQ]; Figure 3C, D and Table S3) and m^5^C‐IP‐seq (Figure 3E, F). Notably, the knockdown of NSUN2 resulted in a significant change in the transcriptome, with 552 downregulated and 268 upregulated genes (Figure 3A). Since most altered genes (552/820, 67.3%) presented decreased expression levels in NSUN2‐deficient cells, our results agree with previous observations that NSUN2‐mediated m^5^C modification maintains RNA stability and thereby enhances gene expression.^17^ Moreover, the altered genes identified are highly related to several events in cancer pathogenesis, including PI3K‐Akt signalling, MAPK signalling and several metabolic pathways (Figure 3B and Figure S4A). Interestingly, proteomic analysis also revealed that NSUN2 gave rise to a salient change in protein expression patterns, which were enriched in proteins associated with glycolysis, metabolism and ribosomes (Figure 3C, D and Figure S4B). Most importantly, we performed genome‐wide m^5^C‐methylated‐RNA‐immunoprecipitation‐sequencing (m^5^C‐meRIP‐seq) to identify m^5^C‐methylated transcripts in both RB cells and normal control cells (Figure 3E). Importantly, m^5^C‐meRIP‐seq revealed a context‐dependent m^5^C modification pattern between RB cells and normal control cells (Figure S5), and their differentially methylated loci were enriched in oncogenesis‐related pathways, including pathways in cancer, Wnt signalling, MAPK signalling, the cell cycle and migration (Figure 3F and Figure S5D). Taken together, these data reveal that NSUN2‐mediated m^5^C modification potentially regulates diverse RNA candidates in the pathogenesis of RB.

**FIGURE 4:**
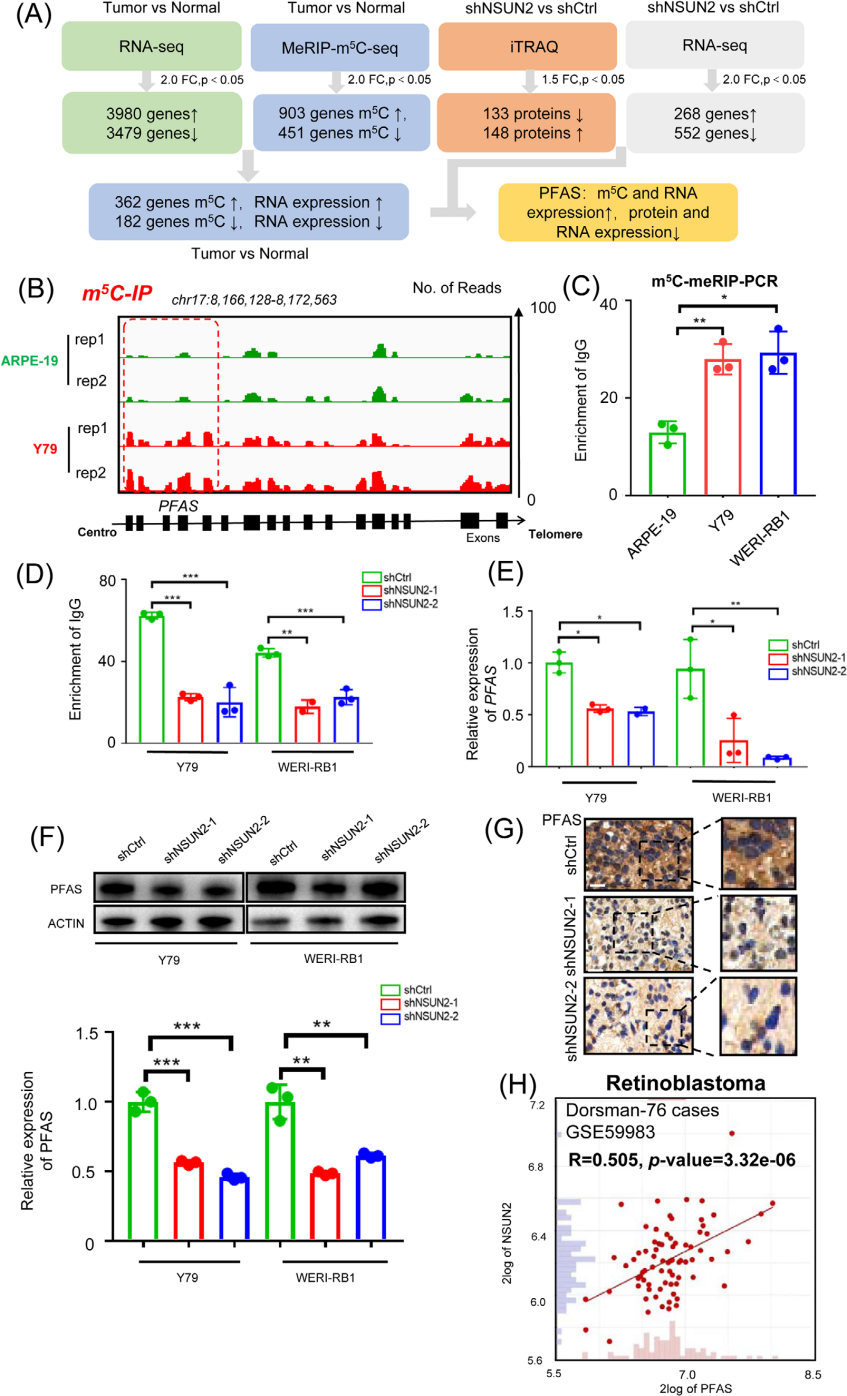
PFAS modulates m^5^C modification of NSUN2. (A) Multiomics analysis identified PFAS as a downstream target of NSUN2. (B) IGV tracks from m^5^C‐meRIP‐seq analysis showing m^5^C enrichment of PFAS. Biological duplicates were analysed. (C) m^5^C‐MeRIP‐qPCR assay of m^5^C status in PFAS in retinoblastoma cells and retinal pigment epithelium cells. The data are presented as the mean ± SD of experimental triplicates. Significance was determined by an unpaired two‐tailed Student's *t* test. *p < .05, **p < .01. (D) m^5^C‐MeRIP‐qPCR assay of m^5^C status in PFAS in NSUN2‐deficient retinoblastoma cells. The data are presented as the mean ± SD of experimental triplicates. Significance was determined by an unpaired two‐tailed Student's *t* test. (E) qPCR data showing PFAS expression in retinoblastoma cells (Y79 and WERI‐RB1) following NSUN2 knockdown. The data are presented as the mean ± SD of experimental triplicates. Significance was determined by an unpaired two‐tailed Student's *t* test. *p < .05, **p < .01. (F) Western blot and statistical analysis showing PFAS expression in retinoblastoma cells (Y79 and WERI‐RB1) following NSUN2 knockdown. The data are representative of experimental triplicates. The data are presented as the mean ± SD of experimental triplicates. Significance was determined by an unpaired two‐tailed Student's *t* test. **p < .01, ***p < .001. (G) Immunohistochemical staining images showing PFAS expression in the control group and NSUN2 knockdown group. Scale bars: left panel, 50 μm; right panel, 25 μm. (H) Correlation analysis of NSUN2 expression and PFAS expression in a cohort of retinoblastoma samples (*n* = 76). Significance was determined by Pearson correlation analysis (*R* = .505, *p* = 3.32e‐06).

To identify the functional target of NSUN2 in RB, we conducted an in‐depth evaluation of the previously performed high‐throughput multiomics analyses. As a result, we found that 362 genes were specifically upregulated in RB cells (Figure 4A, green box, RNA‐seq: tumour vs. normal), with increased m^5^C modification levels (Figure 4B, blue box, meRIP‐m^5^C‐seq: tumour vs. normal). Among these genes, we found that *phosphoribosylformylglycinamidine synthase* (PFAS, Figure S6A), which is a vital enzyme in purine biosynthesis,^37^ was downregulated at both the RNA (Figure 4A, grey box, RNA‐seq: shNSUN2 vs. shCtrl, Figure S6B) and protein (Figure 4A, orange box, iTRAQ: shNSUN2 vs. shCtrl) levels in NSUN2‐deficient RB cells. Notably, PFAS has been reported to function as a canonical oncogenic accelerator, which increases the nucleic acid synthesis required for anabolic cell growth and proliferation during tumorigenesis.^37^ This observation is in perfect alignment with NSUN2‐mediated pro‐oncogenic behaviours in RB.

Importantly, PFAS was hypermethylated in RB cells, as demonstrated by both m^5^C‐meRIP‐seq (Figure 4B) and m^5^C‐meRIP‐PCR (Figure 4C). However, in NSUN2‐deficient cells, the m^5^C level of PFAS was significantly decreased (Figure 4D), following a remarkable downregulation of *PFAS* RNA (Figure 4E) and protein (Figure 4F) levels. Intriguingly, the NSUN2‐silenced orthotopic xenografts presented decreased PFAS expression, indicating that PFAS was consistently regulated by NSUN2 in vivo (Figure 4G). Most importantly, we found a significant positive correlation (*R* = .505, *p* value = 3.32e‐06) between NSUN2 and PFAS expression in a cohort of 76 RB samples (Figure 4H). Taken together, these data support the idea that NSUN2 modulates the PFAS m^5^C modification level and potentially enhances PFAS expression.

### 3.4 PFAS serves as a functional downstream target of NSUN2

**FIGURE 5:**
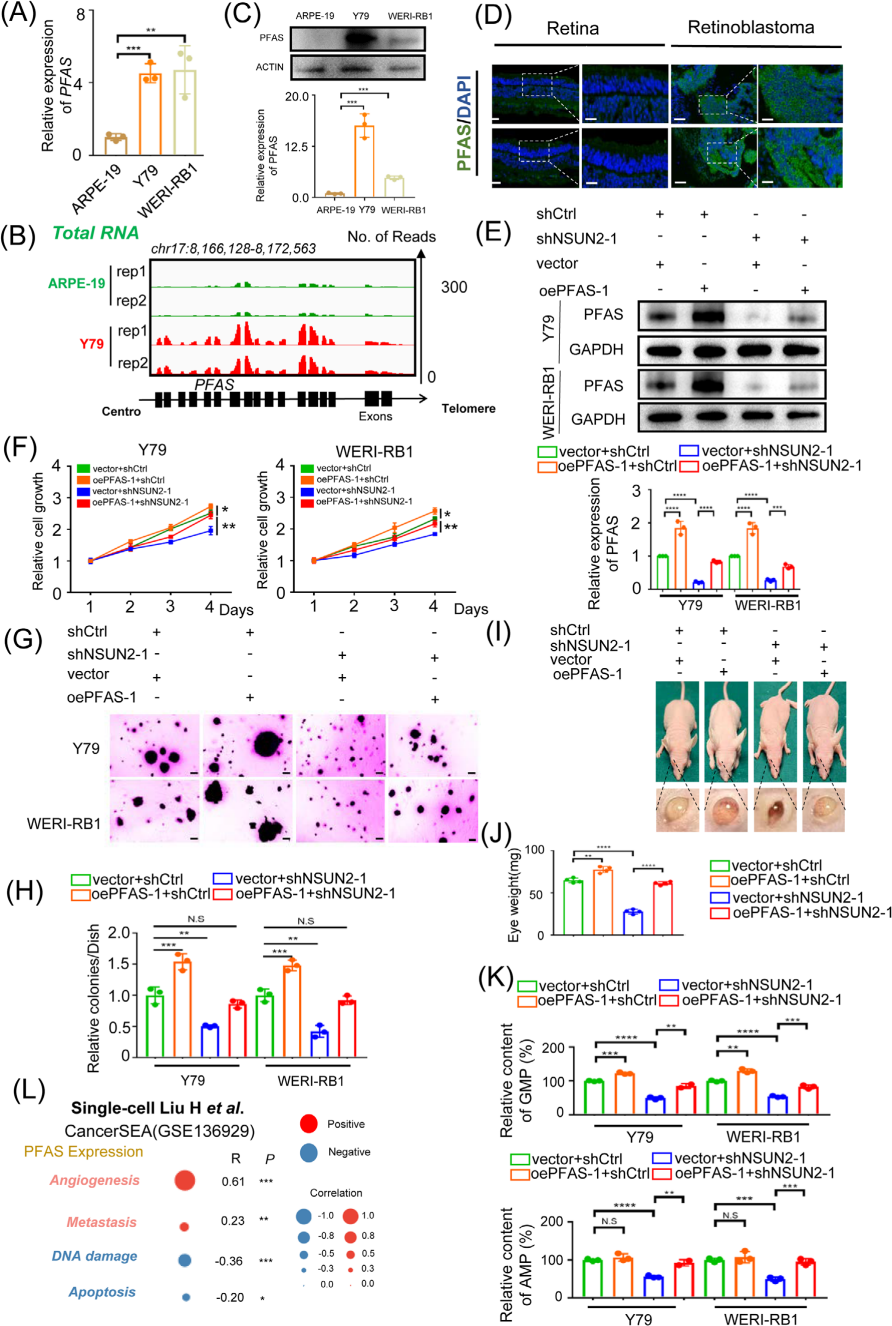
PFAS serves as a functional downstream target of NSUN2. (A) qPCR data showing PFAS expression in retinoblastoma cells relative to that in ARPE‐19 cells. The data are presented as the mean ± SD of experimental triplicates. Significance was determined by an unpaired two‐tailed Student's *t* test. **p < .01, ***p < .001. (B) IGV showing higher expression of PFAS in retinoblastoma relative to normal retina tissue. (C) Western blot data and statistical analysis showing PFAS expression in retinoblastoma cells relative to ARPE‐19 cells. The data are representative of experimental triplicates. The data are presented as the mean ± SD of experimental triplicates. Significance was determined by an unpaired two‐tailed Student's *t* test. ***p < .001. (D) Immunofluorescence of PFAS (green) and DAPI staining (blue) in tumour and normal samples. Scale bars: left panel, 50 μm; right panel, 25 μm. (E) Western blot data and statistical analysis showing PFAS expression in retinoblastoma cells in different groups. The data are representative of experimental triplicates. The data are presented as the mean ± SD of experimental triplicates. Significance was determined by an unpaired two‐tailed Student's *t* test. ***p < .001, **** *p* < .0001. (F) A CCK8 assay was performed to assess the proliferation of NSUN2‐deficient retinoblastoma cells after PFAS overexpression. The data are presented as the mean ± SD of experimental triplicates. Significance was determined by an unpaired two‐tailed Student's *t* test. *p < .05, **p < .01. (G and H) A soft agar assay was performed to assess the proliferation of NSUN2‐deficient retinoblastoma cells following PFAS overexpression. The data are presented as the mean ± SD of experimental triplicates. Significance was determined by an unpaired two‐tailed Student's *t* test. **p < .01, ***p < .001. N.S. indicates no significance. (I and J) Images of BALB/c nude mice and eyeballs containing xenografts derived from NSUN2‐deficient and PFAS‐overexpressed Y79 cells. Statistical analysis of the eyeball weight data. The data are presented as the mean ± SD. Significance was determined by an unpaired two‐tailed Student's *t* test. **p < .01, ****p < .0001. (K) adenosine monophosphate (AMP) and guanosine monophosphate (GMP) concentrations were detected in NSUN2‐deficient retinoblastoma cells following PFAS overexpression. The data are presented as the mean ± SD of experimental triplicates. Significance was determined by an unpaired two‐tailed Student's *t* test. **p < .01, ***p < .001, ****p < .0001. N.S. indicates no significance. (L) Single‐cell transcriptome profiling revealing the correlation between relative PFAS protein expression and different biological processes in retinoblastoma. **p < .01, ***p < .001.

PFAS has been revealed to dictate *de novo* purine synthesis, facilitating malignant cell growth and proliferation by enhancing anabolic RNA and DNA synthesis.^37^ However, the expression level of PFAS in RB remains enigmatic. Consistent with previous high‐throughput analysis, we found that *PFAS* was significantly upregulated at both the RNA (Figure 5A, B) and protein (Figure 5C) levels. Moreover, a significant elevation in PFAS expression was noted in the clinical RB samples compared to normal retinas (Figure 5D). Taken together, these results indicate that PFAS is tumour‐specifically upregulated in RB.

Since the m^5^C modification and expression levels of PFAS were regulated by NSUN2, we next tested whether PFAS serves as an important target for NSUN2‐mediated oncogenic phenotypes by exogenously overexpressing PFAS (Figure 5E). Importantly, the reintroduction of PFAS largely compromised the efficacy of cell growth inhibition in NSUN2‐deficient cells (Figure 5F–H, green and red groups). More importantly, PFAS‐overexpressing cells were much more resistant following NSUN2 deprivation (Figure 5F–H, orange and red group), indicating that PFAS is a necessary downstream target of NSUN2. To further demonstrate the function of signalling cascade of NSUN2‐PFAS in the tumorigenesis of RB, we have tested the tumour proliferative ability in the orthotopic xenograft model by reintroducing PFAS in NSUN2‐deficient cells (Figure 5I and Figure S7). Consistently, reintroducing of PFAS greatly compromised the inhibitory efficacy of NSUN2 depletion (Figure 5J, blue and red group). Moreover, the PFAS‐overexpressed cells are more resistant towards NSUN2 silencing (Figure 5J, orange and red group). Taken together, these data further confirmed that PFAS functions as a necessary downstream candidate of NSUN2‐mediated oncogenic signalling, both in vivo and in vitro. Since PFAS is required for the formation of adenosine monophosphate (AMP) and guanosine monophosphate (GMP) in purine synthesis,^38^ we then detected the abundance of AMP and GMP under our experimental conditions. Intriguingly, NSUN2 silencing led to a significant decrease in AMP and GMP content (Figure 5K, blue group), which was largely rescued by overexpression of PFAS (Figure 5K, red group). PFAS is a vital enzyme in purine biosynthesis, which syntheses IMP to provide the precursor of adenosine triphosphate and guanosine triphosphate^37^ (Figure S8A). Therefore, we have additionally detected the content of IMP, AMP and GMP content after inhibiting PFAS in Y79 cell. As a result, as detected by high‐performance liquid chromatography, the content of IMP was significantly decreased in the PFAS‐deficient cell (Figure S8B). In addition, the AMP and GMP levels were simultaneously reduced after attenuating PFAS expression (Figure S8C). Taken together, these results indicate that PFAS fuels purine synthesis in RB cells, by supporting the content of several intermediate metabolites, including IMP, AMP and GMP. Notably, according to the previous RB organoid model‐based single‐cell analysis,^27^ PFAS was correlated with enhanced angiogenesis (*R* = .61, *p* < .001) and metastasis (*R* = .23, *p* < .01) scores (Figure S9). Taken together, these data indicate that PFAS serves as a functional downstream RNA target of NSUN2.

### 3.5 ALYREF recognizes m5C‐methylated PFAS and promotes its RNA stability

**FIGURE 6:**
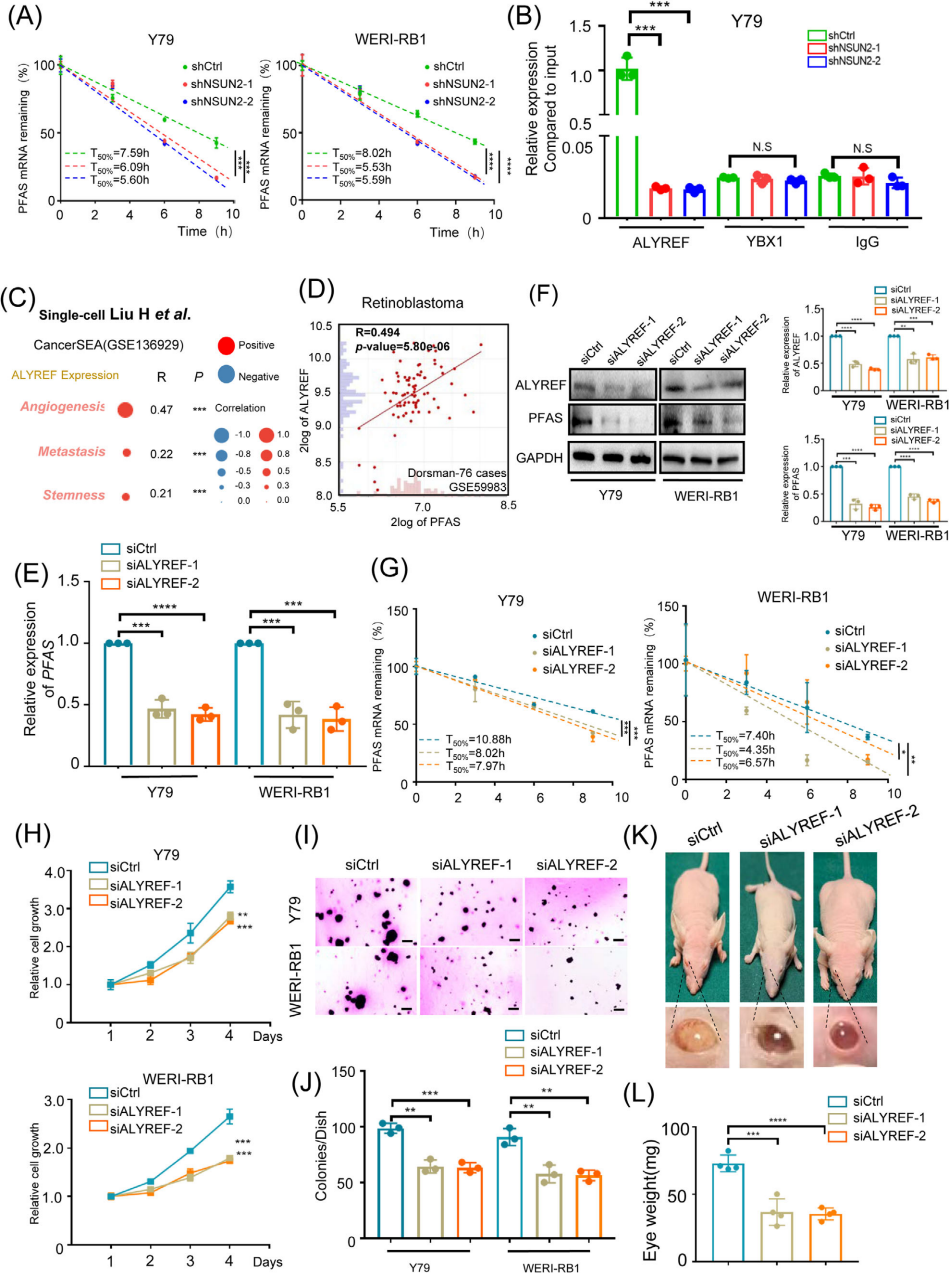
ALYREF recognizes m^5^C‐methylated PFAS and promotes its RNA stability (A) Half‐life of PFAS in NSUN2‐deficient retinoblastoma cells treated with actinomycin (10 μg/μL) for 0–10 h. ***p < .001, ****p < .0001. (B) RIP‐qPCR assay of PFAS expression in retinoblastoma cells (Y79) induced by ALYREF and YBX1. The data are presented as the mean ± SD of experimental triplicates. Significance was determined by an unpaired two‐tailed Student's *t* test. ***p < .001, N.S. indicates no significance. (C) Single‐cell transcriptome profiling revealing the correlation between relative ALYREF protein expression and different biological processes in retinoblastoma. ***p < .001. (D) Correlation analysis of ALYREF expression and PFAS expression in a cohort of retinoblastoma samples (*n* = 76). Significance was determined by Pearson correlation analysis (*R* = .494, *p* = 5.80e‐06). (E) qPCR data showing PFAS expression in retinoblastoma cells (Y79 and WERI‐RB1) following ALYREF knockdown. The data are presented as the mean ± SD of experimental triplicates. Significance was determined by an unpaired two‐tailed Student's *t* test. ** *p* < .01, ***p < .001, ****p < .0001. (F) Western blot and statistical analysis showing PFAS expression in retinoblastoma cells (Y79 and WERI‐RB1) following ALYREF knockdown. The data are representative of experimental triplicates. The data are presented as the mean ± SD of experimental triplicates. Significance was determined by an unpaired two‐tailed Student's *t* test. ***p < .001, **** *p* < .0001. (G) Half‐life of PFAS in ALYREF‐deficient retinoblastoma cells treated with actinomycin (10 mg/mL) for 0–10 h. *p < .05, **p < .01, ***p < .001. (H) A CCK‐8 assay was employed to evaluate the proliferation of retinoblastoma cells (Y79 and WERI‐RB1) after ALYREF knockdown. The data are presented as the mean ± SD of experimental triplicates. Significance was determined by an unpaired two‐tailed Student's *t* test. **p < .01, ***p < .001. (I and J) A soft agar assay was employed to evaluate the proliferation of retinoblastoma cells (Y79 and WERI‐RB1) following ALYREF knockdown. Representative images from three experimental replicates are shown. The data are presented as the mean ± SD. Significance was determined by an unpaired two‐tailed Student's *t* test. **p < .01, ***p < .001. (K and L) Images of BALB/c nude mice and eyeballs containing xenografts derived from ALYREF‐deficient Y79 cells. Statistical analysis of the eyeball weight data. The data are presented as the mean ± SD. Significance was determined by an unpaired two‐tailed Student's *t* test. ***p < .001, ****p < .0001.

We then explored the detailed mechanism underlying the m^5^C regulation of PFAS by NSUN2. Since m^5^C modifications have been revealed to play vital roles in the maintenance of RNA stability,^17^ we first tested whether NSUN2 regulates the RNA stability of PFAS. As a result, we found that the RNA stability of PFAS was dramatically decreased in NSUN2‐deficient cells (Figure 6A), which is in alignment with previous observations that NSUN2 promotes PFAS expression. Importantly, YBX1 and ALYREF have been revealed as important m^5^C readers, increasing the RNA stability of diverse RNA targets.^18^, ^39^ We then tested whether YBX1 and ALYREF can potentially interact with the PFAS transcript. We found that ALYREF significantly interacted with PFAS; however, the ALYREF−PFAS interaction was diminished after NSUN2 inhibition, which indicates that the ALYREF−PFAS interaction requires m^5^C modification. Notably, YBX1 showed only a limited interaction signal, which was similar to that in the negative control (IgG) group (Figure 6B). Taken together, these data indicate that ALYREF serves as an important recognition protein for m^5^C‐methylated PFAS mRNA. Notably, according to the previous RB organoid model‐based single‐cell analysis,^27^ ALYREF is also correlated with enhanced angiogenesis (*R* = .47, *p* < .001), metastasis (*R* = .22, *p* < .001) and stemness (*R* = .21, *p* < .001) scores (Figure 6C and Figure S10). Most importantly, ALYREF presented parallel expression with PFAS (*R* = .494, *p* = 5.80e‐06, Figure 6D and Figure S11), further indicating that ALYREF participates in the NSUN2/PFAS oncogenic cascade during RB progression.

To verify the importance of the regulation of PFAS by ALYREF, we subsequently silenced ALYREF using two individual siRNAs and assessed the expression levels of PFAS. Intriguingly, the mRNA (Figure 6E) and protein (Figure 6F) levels of *PFAS* were markedly decreased after ALYREF silencing, followed by a significant reduction in RNA stability (Figure 6G). In addition, we have further tested the function of ALYREF in the tumorigenesis of RB, both in vitro and in vivo. Notably, ALYREF silencing led to a remarkably decreased proliferation rate (Figure 6H) and attenuated colony formation capacity (Figure 6I, J) in RB cells. Moreover, the silencing of ALYREF also contributes to a decreased tumour formation ability in the orthotopic xenografts (Figure 6K, L and Figure S12). Since ALYREF is responsible for the recognition of PFAS, these observations are in alignment with the decreased expression of PFAS upon ALYREF silencing. ALYREF is considered as a nucleus m^5^C reader to recognize and facilitate RNA transportation of m^5^C modified mRNA.^39^ Moreover, ALYREF has been revealed to promote RNA stability through competing with hMTR4, which is responsible for RNA degradation of nuclear transcripts.^40^ We then detected the proportion of PFAS RNA in nucleus (labelled by U6) and cytoplasm (labelled by GAPDH). Interestingly, the proportion of PFAS RNA in nucleus was remarkably increased in ALYREF‐deficient RB cells, while the distribution pattern of GAPDH and U6 remains unchanged (Figure S13A). Concordantly, we observed increased interaction frequency between hMTR4 and PFAS transcript (Figure S13B), which agrees with a competing mechanism between ALYREF and hMTR4.^40^ Since RNA stability of PFAS was dramatically decreased in NSUN2‐deficient cells and hMTR4 has been revealed as an important nuclear factor for RNA degradation, our results aggregate the fact that ALYREF‐bound PFAS transcript prevents its binding with hMTR4, which gives rise to increased mRNA stability in an m^5^C‐dependent manner. Taken together, these results support the idea that ALYREF serves as an important recognition regulator of the m^5^C‐methylated PFAS transcript.

## 4 DISCUSSION

**FIGURE 7:**
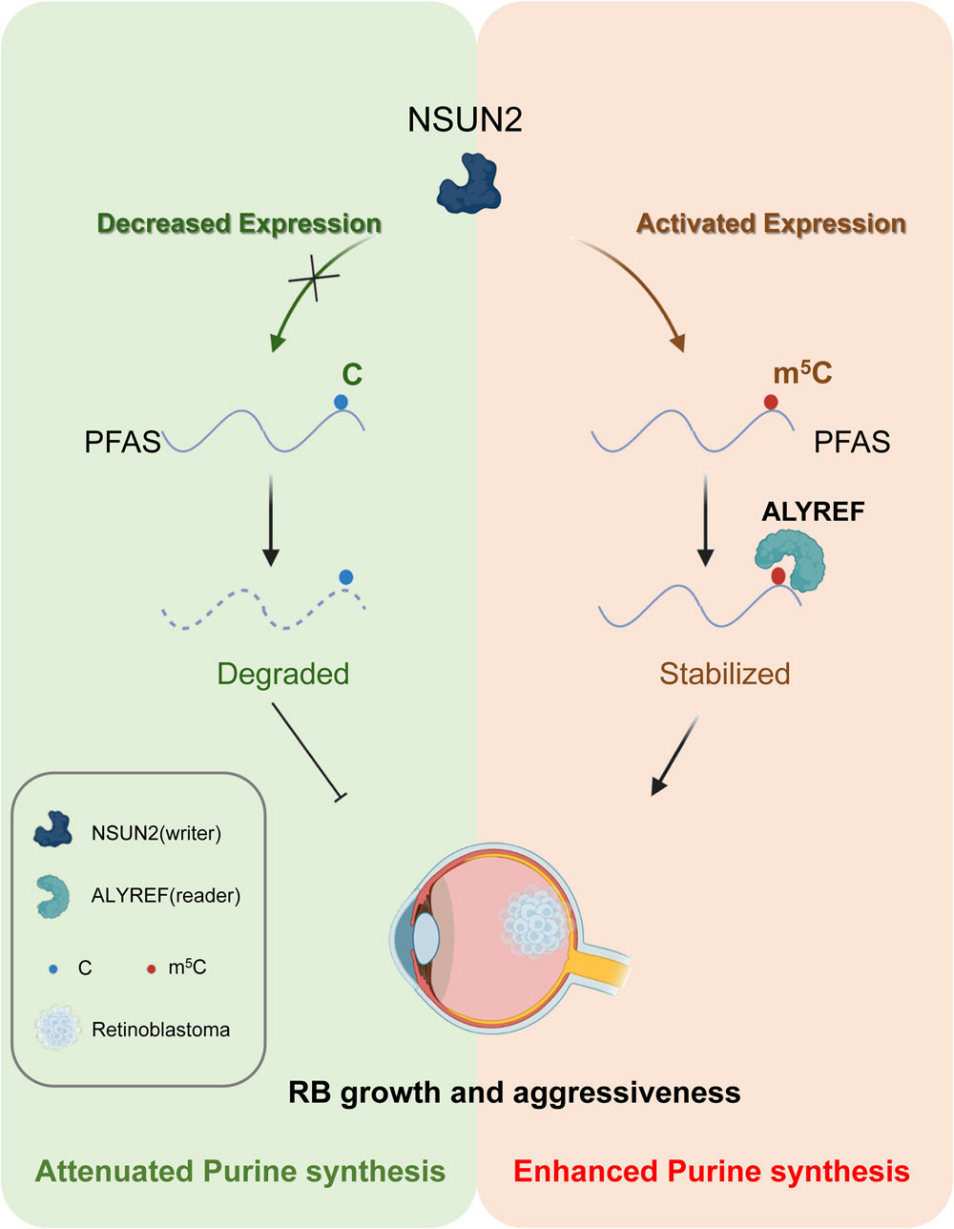
Schematic diagram of this study. Enhancement of m^5^C methylation induced by NSUN2 upregulation in retinoblastoma cells leads to increased PFAS mRNA and protein expression and higher adenosine monophosphate (AMP) and guanosine monophosphate (GMP) content, which contribute to the aggressiveness related to retinoblastoma progression.

Dynamic and reversible regulation of m^5^C RNA modification plays essential roles in the determination of RNA fate, controlling RNA stability and thereby regulating its expression.^41^, ^42^, ^43^, ^44^ RB is the most frequent intraocular malignancy in childhood, causing visual loss and even death.^20^, ^45^ However, the relationship between m^5^C RNA modification and RB remains enigmatic. In this study, we demonstrated that NSUN2‐mediated m^5^C RNA methylation was tumour‐specifically elevated, which fuels purine biosynthesis during the oncogenic progression of RB. Mechanistically, NSUN2 methylated the PFAS transcript and promoted its RNA stability in an ALYREF‐dependent manner. Conclusively, this study initially demonstrated that NSUN2 is necessary for oncogenic gene activation in RB, expanding the current understanding of dynamic m^5^C function during tumour progression. Since NSUN2‐mediated m^5^C RNA modification is an important trigger of purine synthesis, our study bridges the current knowledge of m^5^C modification and metabolic reprogramming in cancer (Figure 7).

Notably, NSUN2, a 5‐methylcytosine RNA methyltransferase, has been reported to function as an indicative regulator of the cell proliferation and metastasis observed in diverse cancer types. For example, NSUN2‐mediated m^5^C modification promotes the pathogenesis of bladder cancer by enhancing the binding of YBX1 to HDGF and thereby promotes its expression.^18^ Moreover, NSUN2 fuels gastric cancer cell proliferation by repressing CDKN1C in an m^5^C‐dependent manner.^46^ In addition, NSUN2 activates receptor‐bound protein 2 (GRB2) and subsequently accelerates ESCC.^17^ In the present study, we found that NSUN2 also serves as an important oncogenic promoter in RB, expanding the current understanding of dynamic m^5^C function during tumour progression. These studies indicated that NSUN2‐mediated m^5^C modification regulates cancer cell fate through the metabolism of mRNA. Moreover, recent studies have demonstrated that m^5^C RNA modifications of rRNA and tRNA participate in diversified biological functions. For instance, NSUN1‐mediated m^5^C regulates ribosome biogenesis via regulating pre‐rRNA processing.^47^ Moreover, m^5^C modification regulates tRNA function and the brain proteome to cognition and complex behaviours.^48^ In addition, NSUN2‐driven tRNA methylation was functionally required to adapt cell cycle progression to the early stress response.^49^ Since m^5^C modification could also regulate metabolic reprogramming through tRNA methylation, the role of m^5^C‐mediated tRNA methylation in RB tumorigenesis awaits successive investigations.

Notably, PFAS is an essential enzyme in the *de novo* synthesis of purines, playing an important role in RIG‐I activation during viral infection, autophagic regulation and carcinogenesis.^50^, ^51^, ^52^ For example, a genome‐wide CRISPR screen revealed that PFAS regulates mTOR activity and thereby inhibits autophagic flux.^51^ Moreover, PFAS also participates in ERK signalling and supports anabolic cell growth by increasing DNA/RNA synthesis.^37^ In this study, we report for the first time that PFAS serves as an oncogenic promoter in RB and is a potential RNA target recognized by NSUN2.

## 5 CONCLUSIONS

In this study, we initially demonstrated that NSUN2 is necessary for PFAS activation by enhancing its RNA stability in RB, which expands the current understanding of dynamic m^5^C function during tumour progression. Notably, we revealed that m^5^C modification on RNA is important for purine synthesis, which bridges the current understanding of RNA modification and metabolic reprogramming. Since the NSUN2/ALYREF/m^5^C‐PFAS oncogenic cascade is an important trigger of RB, our study provides a novel therapeutic strategy, namely, a ‘targeted m^5^C reprogramming strategy’, that may potentially be an efficient anti‐tumour therapy.

## CONFLICT OF INTEREST STATEMENT

The authors declare that they have no conflict of interest.

## Supporting information

Supporting Information
