# Supplementary material for: NSUN2‐mediated m5C RNA methylation dictates retinoblastoma progression through promoting PFAS mRNA stability and expression
Source: Clin Transl Med. 2023 May 25;13(5):e1273. doi: 10.1002/ctm2.1273 (PMC10212275; doi:10.1002/ctm2.1273)
Supplement: Supplementary file 1 — Supporting Information [file CTM2-13-e1273-s001.docx]

**Sup files**

**NSUN2-mediated m^5^C RNA methylation dictates retinoblastoma progression through promoting PFAS mRNA stability and expression**

Sipeng Zuo^1,2,#^; Lin Li^1,2,#^; Xuyang Wen^1,2,#^; Xiang Gu^1,2^; Ai Zhuang^1,2^; Rui Li^1,2^; Fuxiang Ye^1,2^; Shengfang Ge^1,2^; Xianqun Fan^1,2^; Jiayan Fan^1,2,*^; Peiwei Chai^1,2,*^; Linna Lu^1,2,*^

^1^Department of Ophthalmology, Ninth People's Hospital, Shanghai Jiao Tong University School of Medicine, Shanghai, 200011, People's Republic of China.

^2^Shanghai Key Laboratory of Orbital Diseases and Ocular Oncology, Shanghai, 200011, People's Republic of China.

#These authors contributed equally to this manuscript.

**Correspondence**: Jiayan Fan M.D. & Ph.D., Chai Peiwei, M.D. & Ph.D., Linna Lu, M.D. & Ph.D., Ninth People’s Hospital, Shanghai JiaoTong University School of Medicine, Shanghai, 200025, P.R. China, E-mail: fanjiayan1118@126.com (J.F.); chaipeiwei123@sjtu.edu.cn (P.C.); drlulinna@126.com (L.L.).

**Supplementary files include 15 figures and 3 tables.**

**Supplementary Figures & Legends**


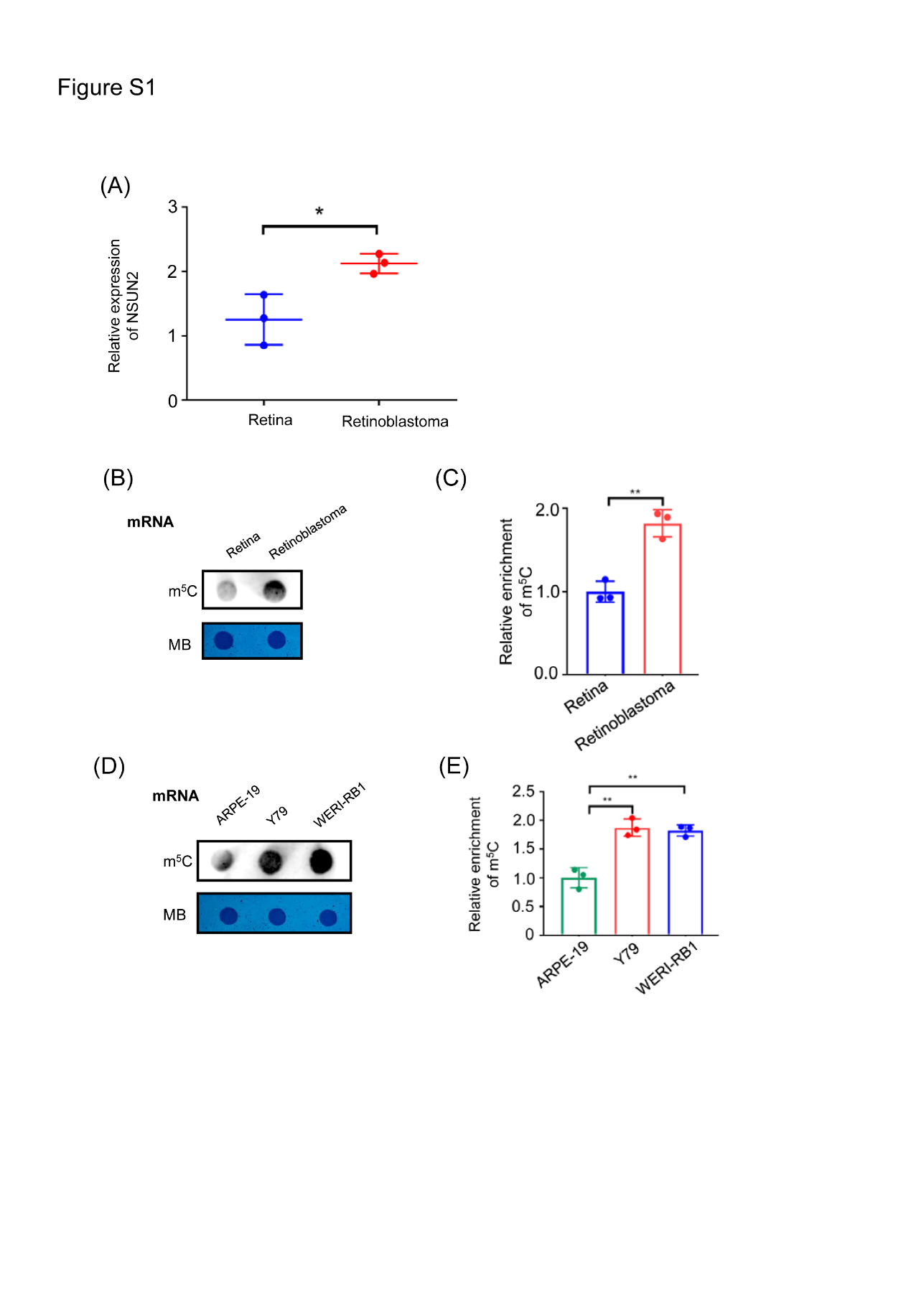


**FIGURE S1.** **The m^5^C modification level increased in retinoblastoma.**

(A) Statistical result of NSUN2 level in normal and tumor tissues. Significance was determined by an unpaired two-tailed Student’s t test. *P < 0.05.

(B and C) Dot blot showing the mRNA m^5^C signal compared to the methylene blue signal in retinoblastoma and normal retina tissue. The data are presented as the mean ± SD of experimental triplicates. Significance was determined by an unpaired two-tailed Student’s t test. **P < 0.01.

(D and E) Dot blot showing the mRNA m^5^C signal compared to the methylene blue signal in retinoblastoma cell lines and retinal pigment epithelium cell lines. The data are presented as the mean ± SD of experimental triplicates. Significance was determined by an unpaired two-tailed Student’s t test. **P < 0.01.

**
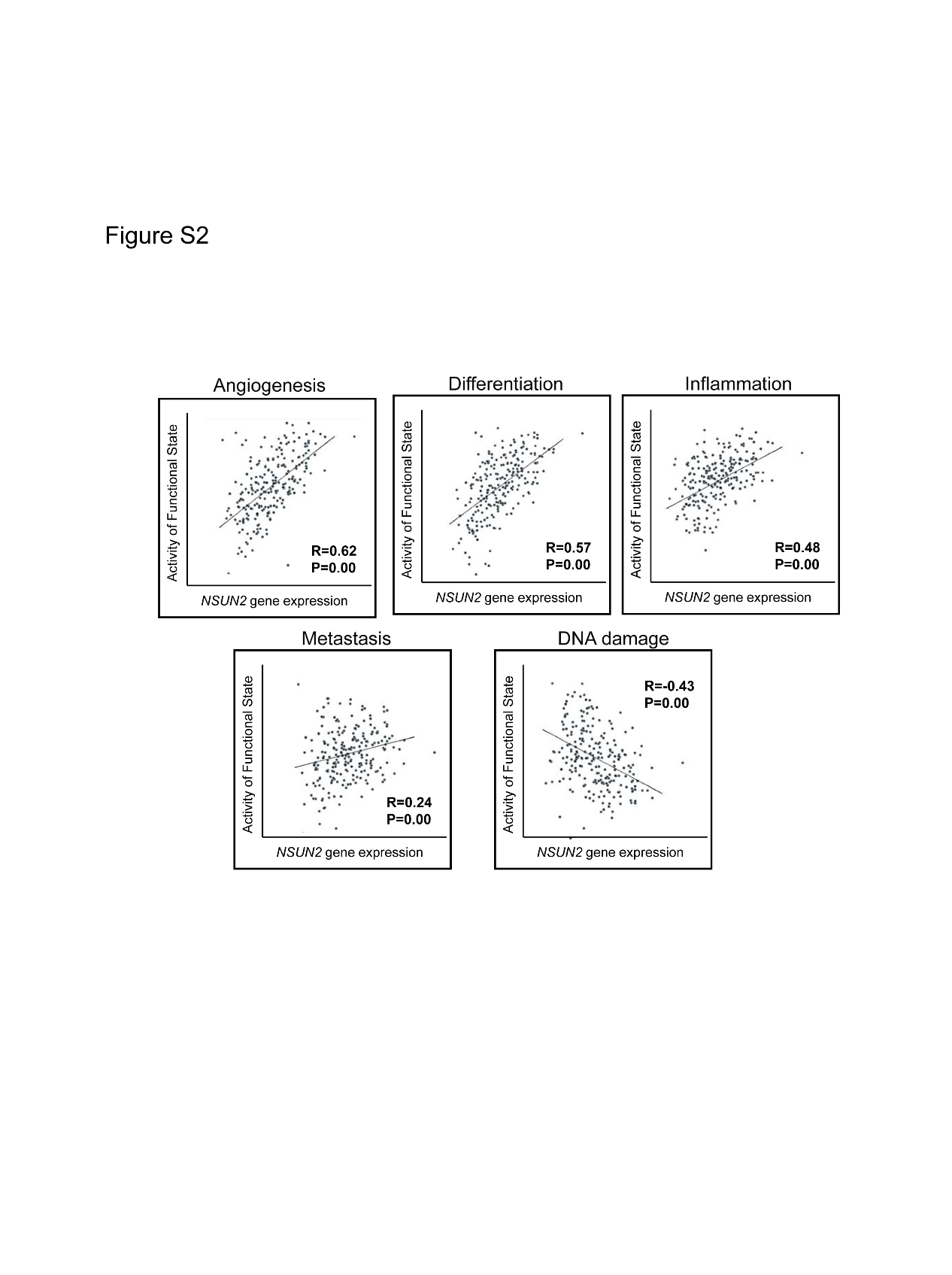
**

**FIGURE S2.** **Single-cell analysis revealed tumor oncogenic functions of NSUN2 in retinoblastoma.**

Single-cell transcriptome profiling revealing the correlation of the relative protein expression of NSUN2 and different functional states (angiogenesis, differentiation, inflammation, metastasis and DNA damage) in retinoblastoma. Significance was determined by Pearson correlation analysis (p< 0.001).


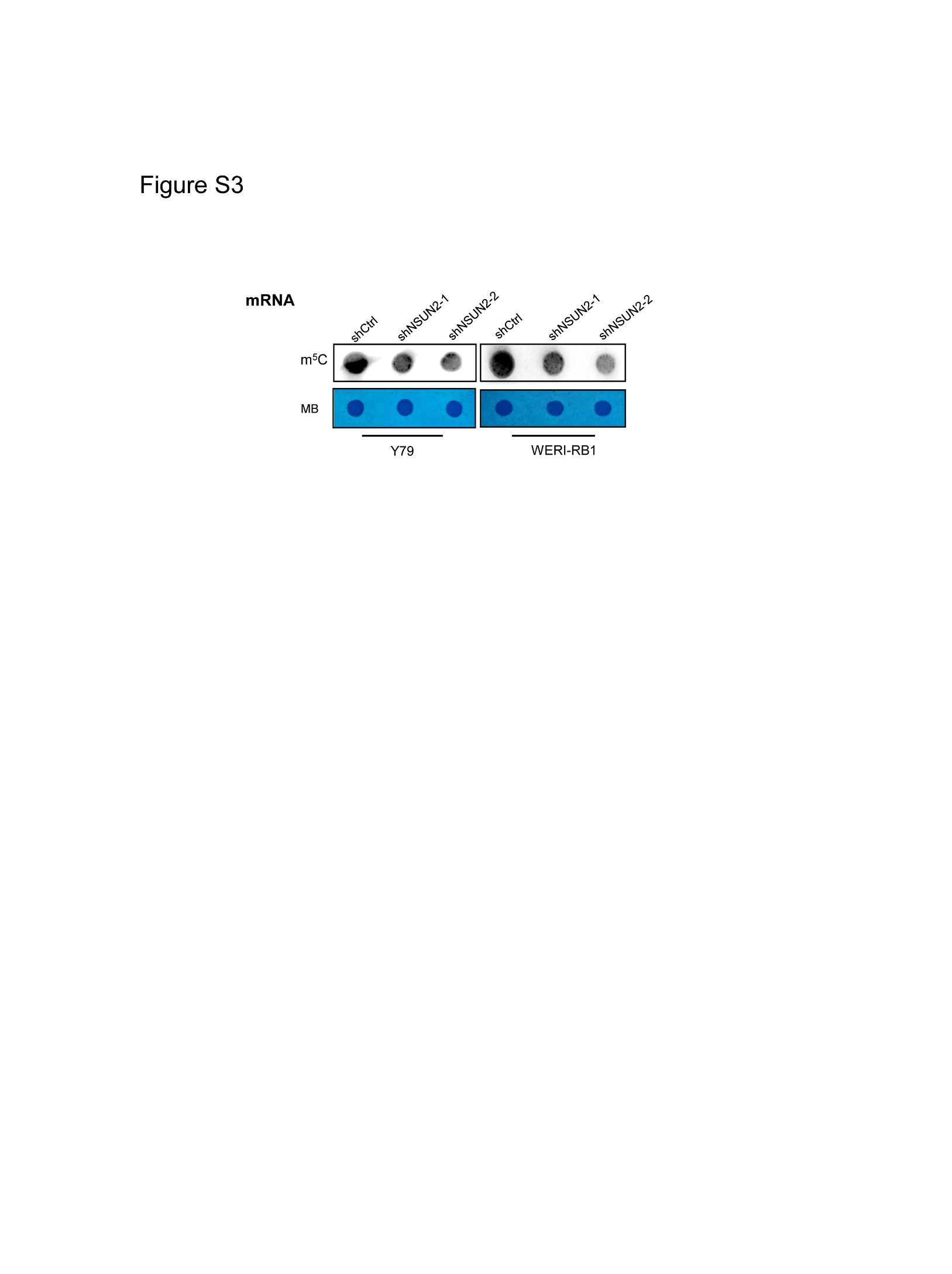


**FIGURE S3. The mRNA m^5^C level decreased following NSUN2 knockdown.**

Dot blot showing the mRNA m^5^C signal relative to the methylene blue signal in retinoblastoma cells (Y79 and WERI-RB1) following NSUN2 knockdown.


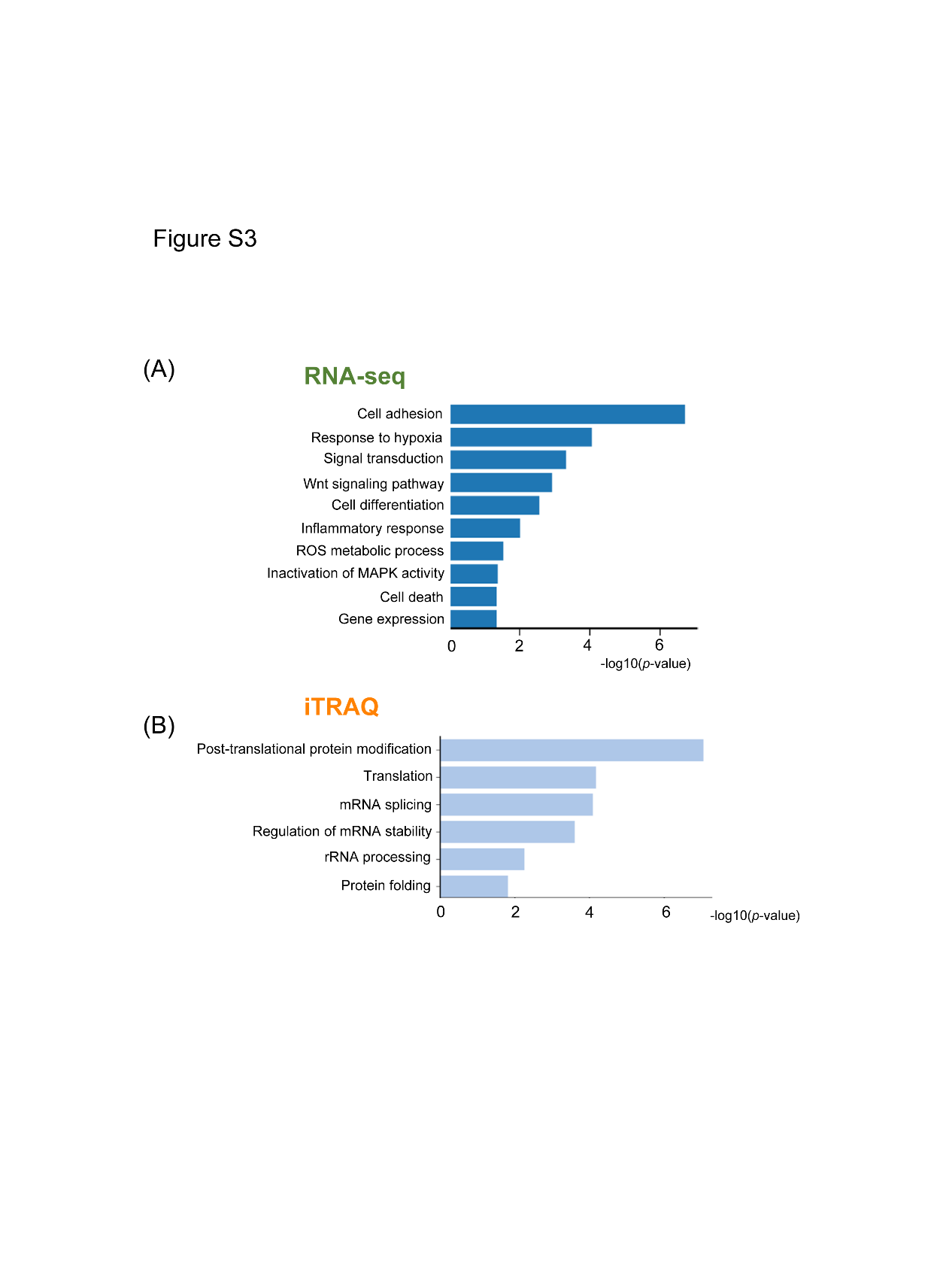


**FIGURE S4.** **Multiomics analysis in NSUN2-deficient retinoblastoma cells.**

(A) Gene ontology (GO) analysis of NSUN2-regulated genes in NSUN2-deficient retinoblastoma cells.

(B) GO analysis of NSUN2-regulated proteins in NSUN2-deficient retinoblastoma cells.


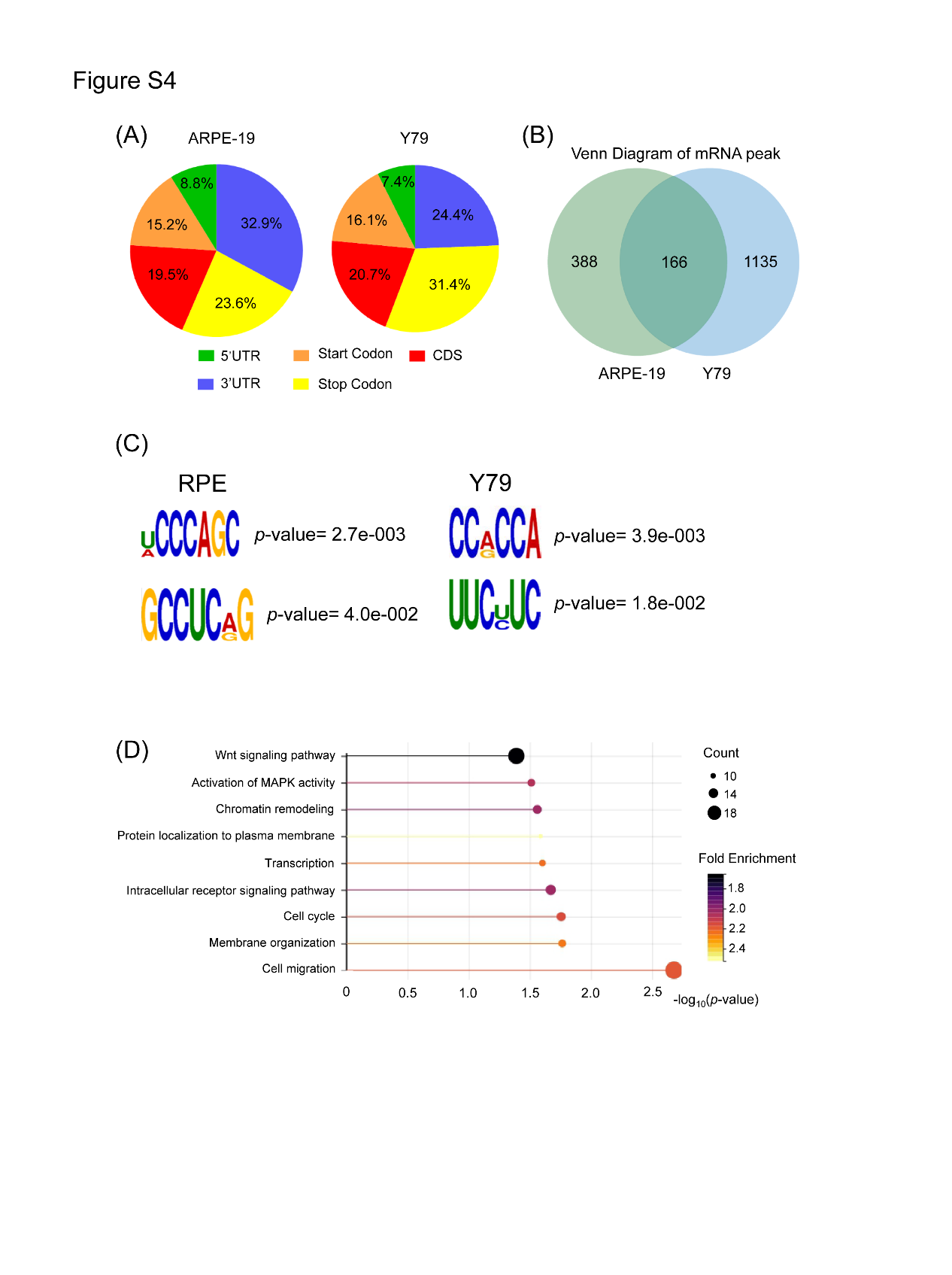


**FIGURE S5. Dynamic m^5^C modifications in retinoblastoma.**

(A) Pie charts showing the m^5^C peak distribution in different RNA regions (CDS, 5′ UTR, 3′ UTR, start codon and stop codon) in retinoblastoma cells and normal retinal pigment epithelium cells.

(B) Venn diagram showing the number of m^5^C peak distribution in mRNA in retinoblastoma cells and normal retinal pigment epithelium cells.

(C) Top enriched motifs within m^5^C peaks identified in retinoblastoma cells and normal retinal pigment epithelium cells.

(D) KEGG analysis of m^5^C-regulated genes in retinoblastoma cells and normal retinal pigment epithelium cells.


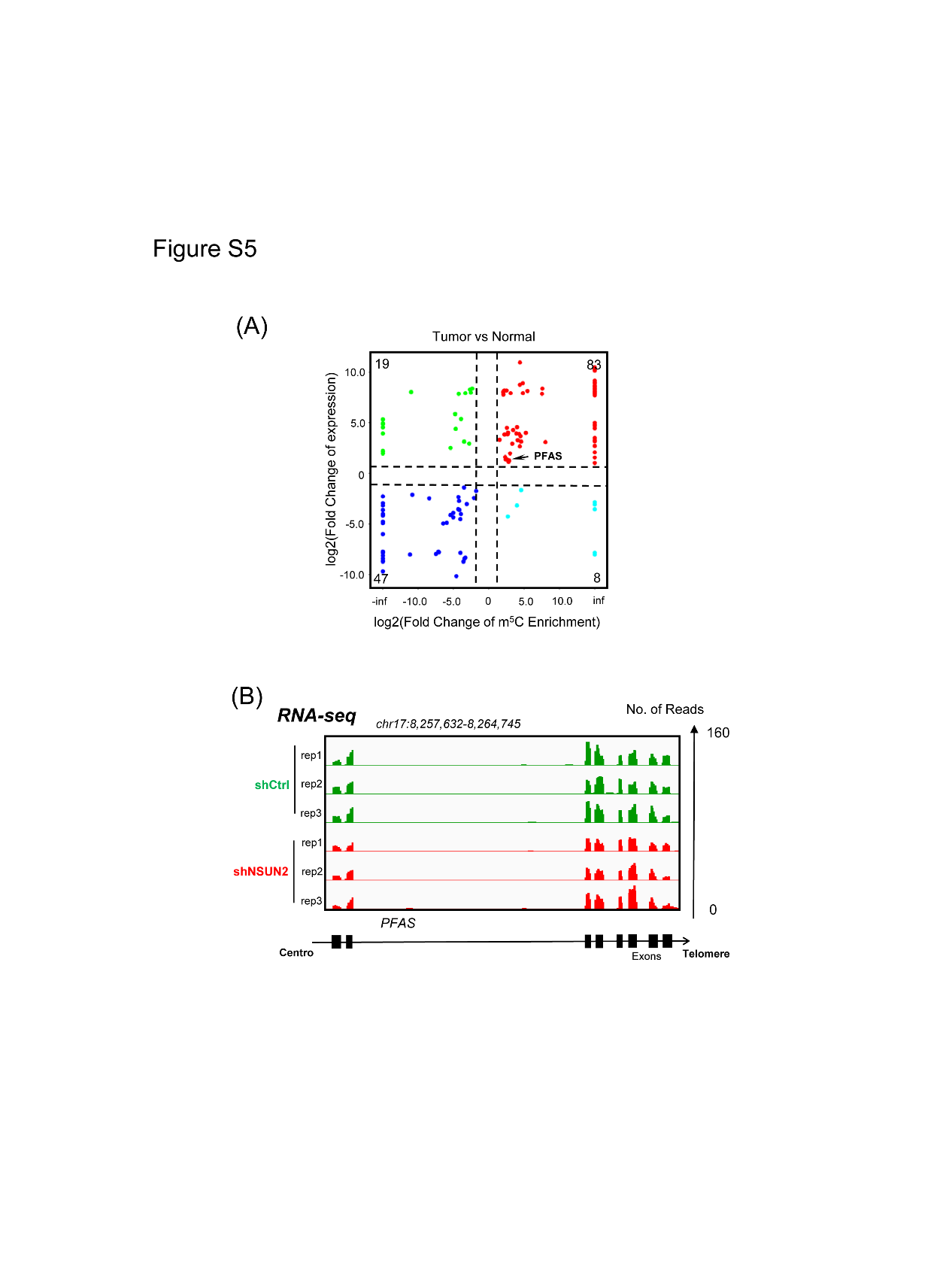


**FIGURE S6.** **Correlation of PFAS with m^5^C and NSUN2.**

(A) Volcano plot showing the m^5^C enrichment and genes expression in normal cells compared to tumor cells.

(B) IGV showing the expression of PFAS in retinoblastoma cells (Y79) upon NSUN2 knockdown.


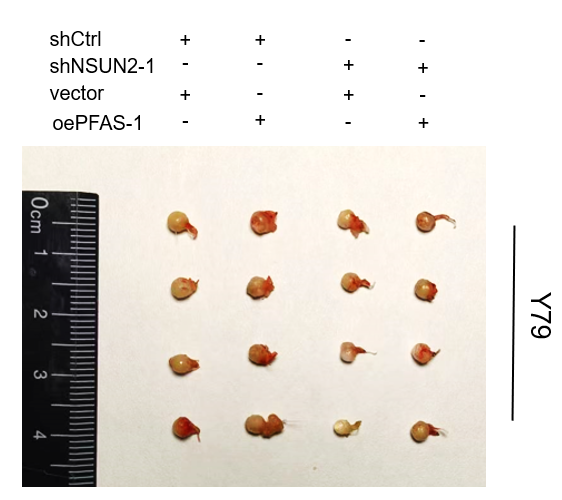


**FIGURE S7.** **PFAS serves as a functional downstream target of NSUN2.**

Images of eyeballs containing xenografts derived from NSUN2-deficient and PFAS-overexpressed Y79 cells. Representative images from four biological replicates are shown.


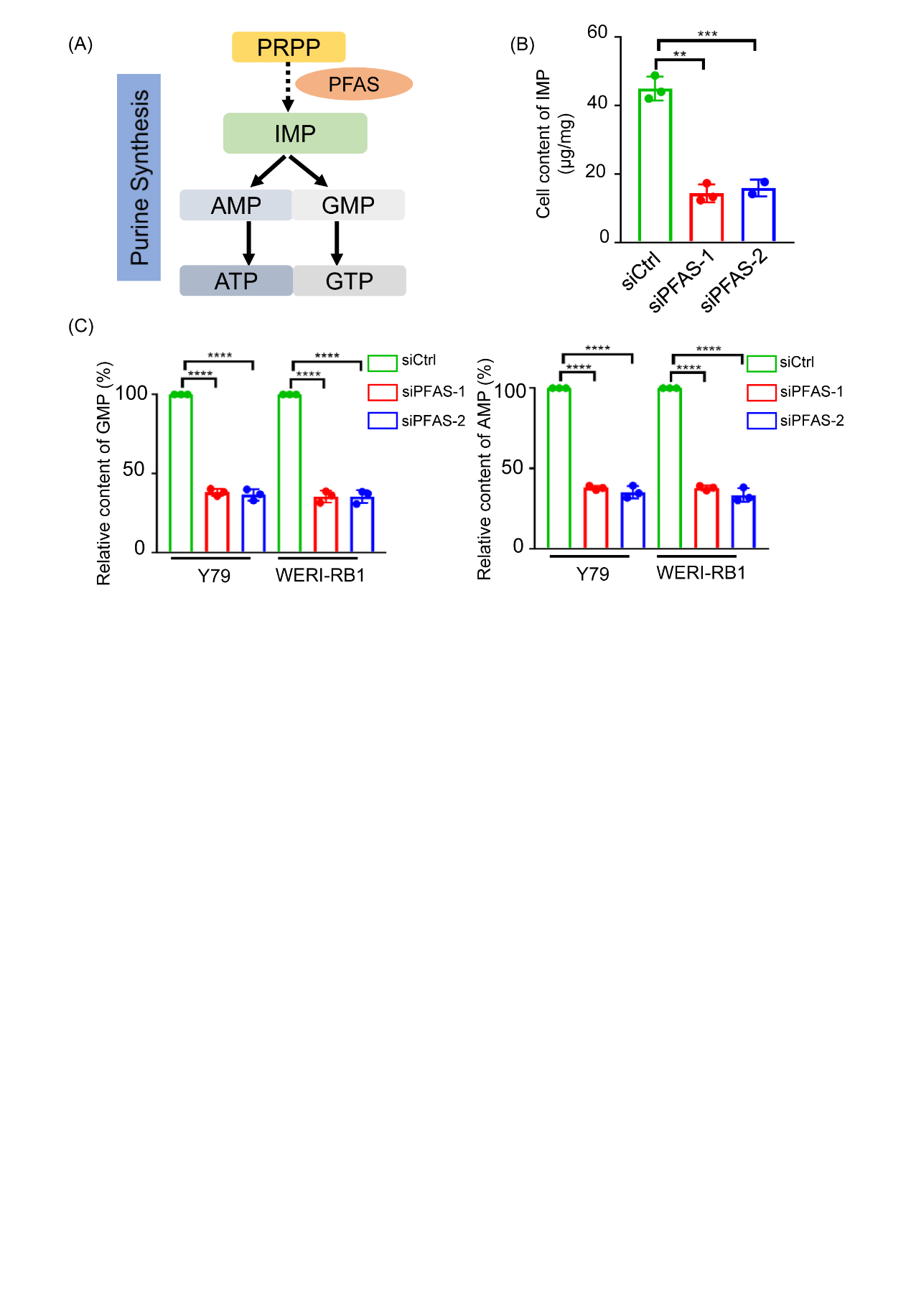


**FIGURE S8.** **PFAS fuels purine synthesis in retinoblastoma cells.**

(A) An outline of purine synthesis.

(B) High Performance Liquid Chromatography (HPLC) showing the content of IMP in retinoblastoma cells (Y79) following PFAS knockdown. The data are presented as the mean ± SD of experimental replications. Significance was determined by an unpaired two-tailed Student’s t test. **P<0.01, ***P<0.001.

(C) AMP and GMP concentrations were detected in PFAS-deficient retinoblastoma cells. The data are presented as the mean ± SD of experimental triplicates. Significance was determined by an unpaired two-tailed Student’s t test. ****P < 0.0001.


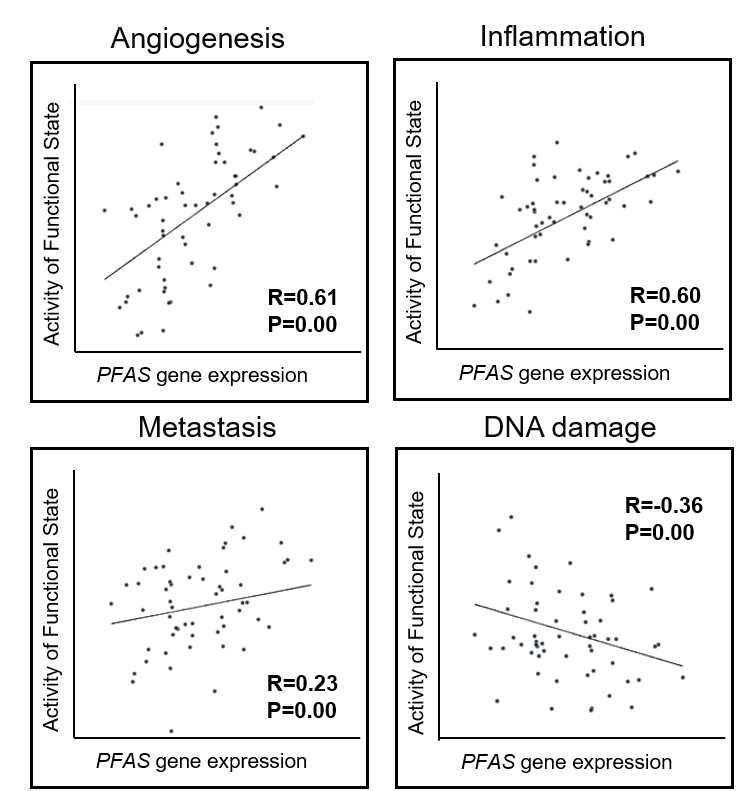


**FIGURE S9. Tumor oncogenic functions of PFAS in retinoblastoma by single-cell analysis.**

Single-cell transcriptome profiling revealing the correlation of the relative protein expression of PFAS and different functional states (angiogenesis, inflammation, metastasis and DNA damage) in retinoblastoma. Significance was determined by Pearson correlation analysis (p< 0.001).


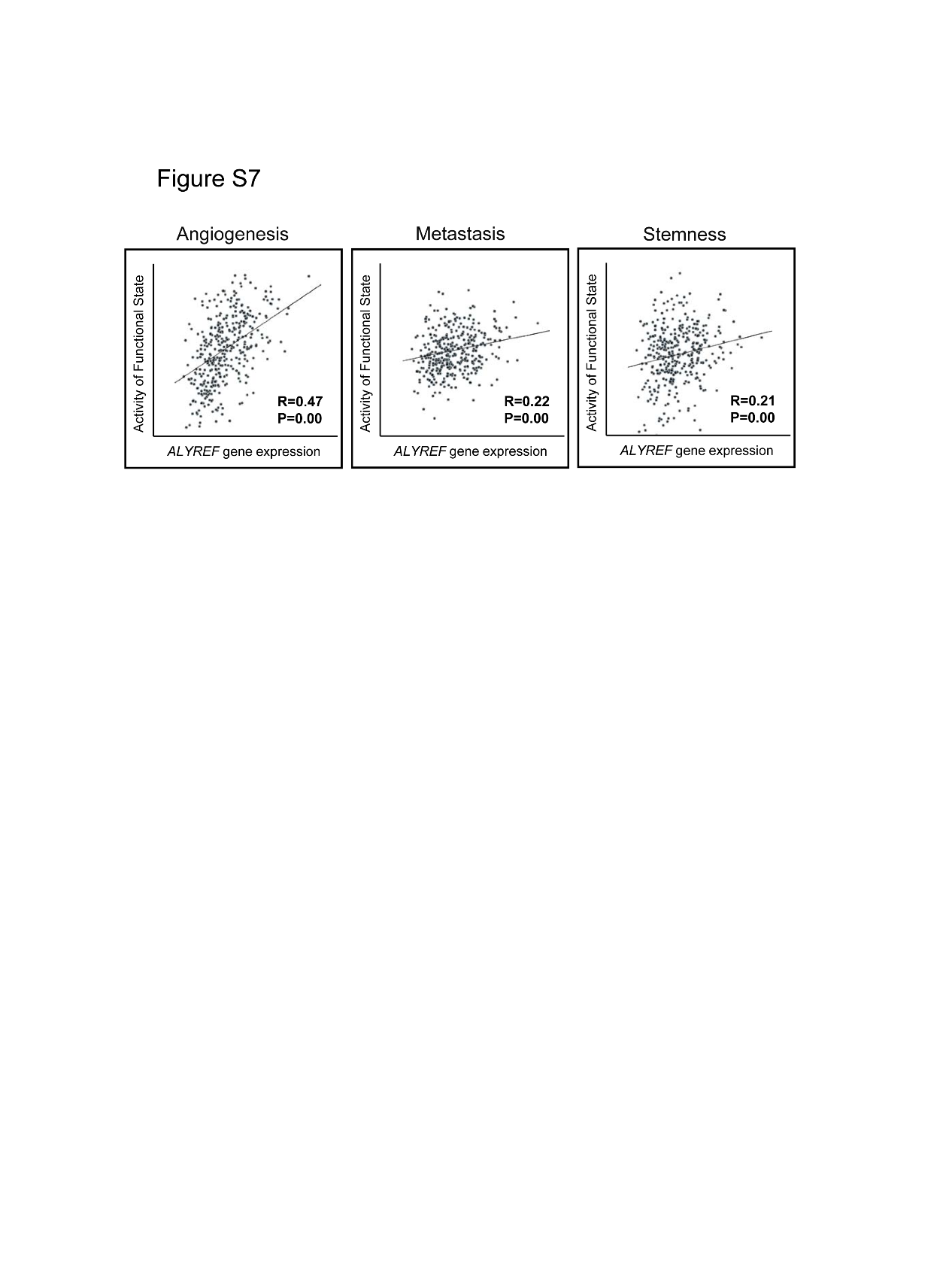


**FIGURE S10.** **Tumor oncogenic functions of ALYREF in retinoblastoma by single-cell analysis.**

Single-cell transcriptome profiling revealing the correlation of the relative protein expression of ALYREF and different functional states (angiogenesis, metastasis and stemness) in retinoblastoma. Significance was determined by Pearson correlation analysis (p< 0.001).


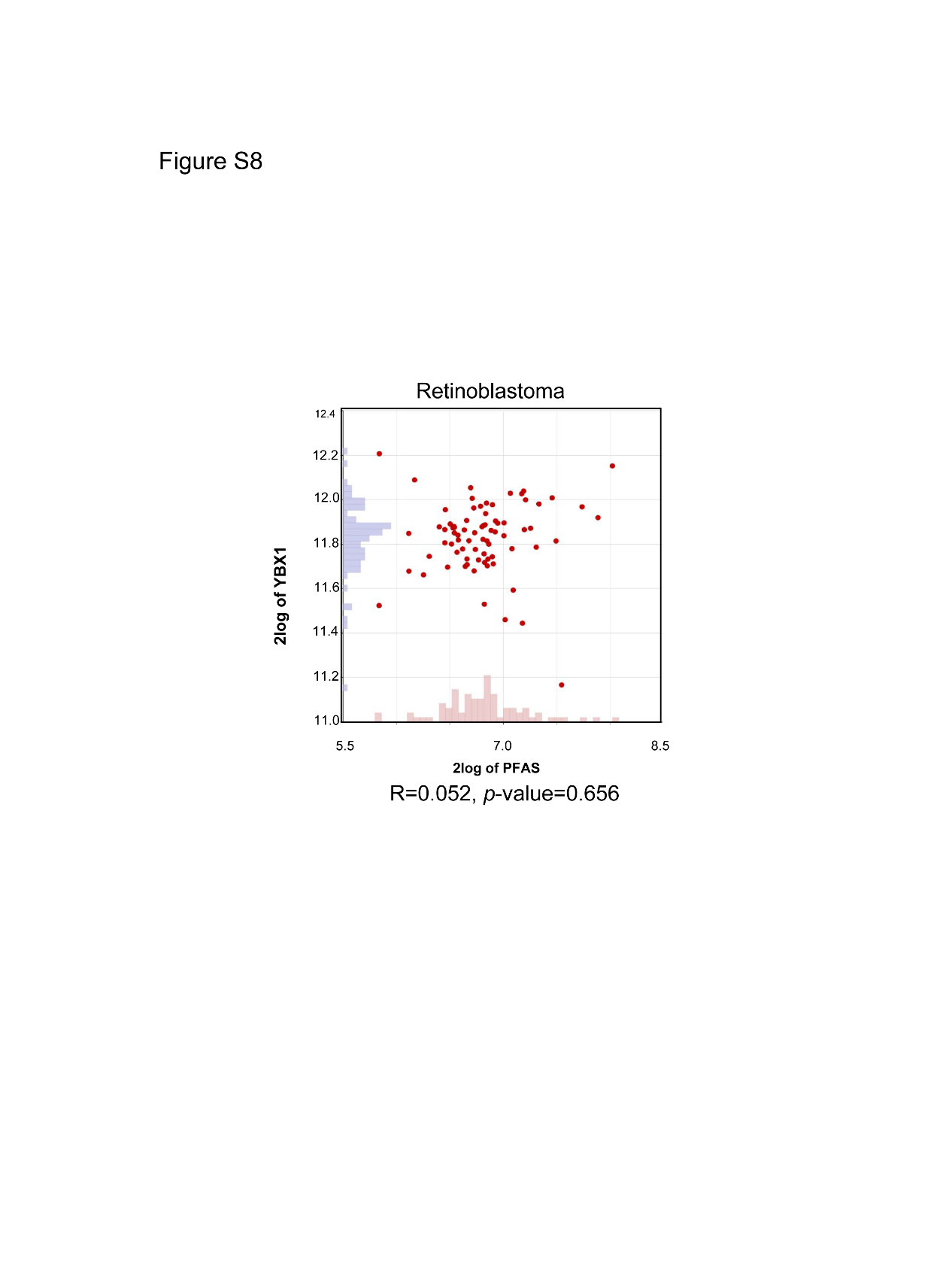


**FIGURE S11. Correlation analysis of YBX1 and PFAS.**

Correlation analysis of YBX1 expression and PFAS expression in a cohort of retinoblastoma samples (n=76). Significance was determined by Pearson

correlation analysis (R = 0.052, P = 0.656).

**
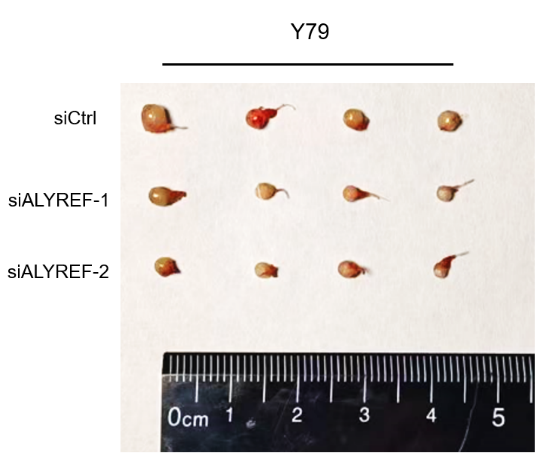
**

**FIGURE S12.** **ALYREF promotes malignant proliferation of retinoblastoma *in vivo*.**

Images of eyeballs containing xenografts derived from ALYREF-deficient Y79 cells. Representative images from four biological replicates are shown.


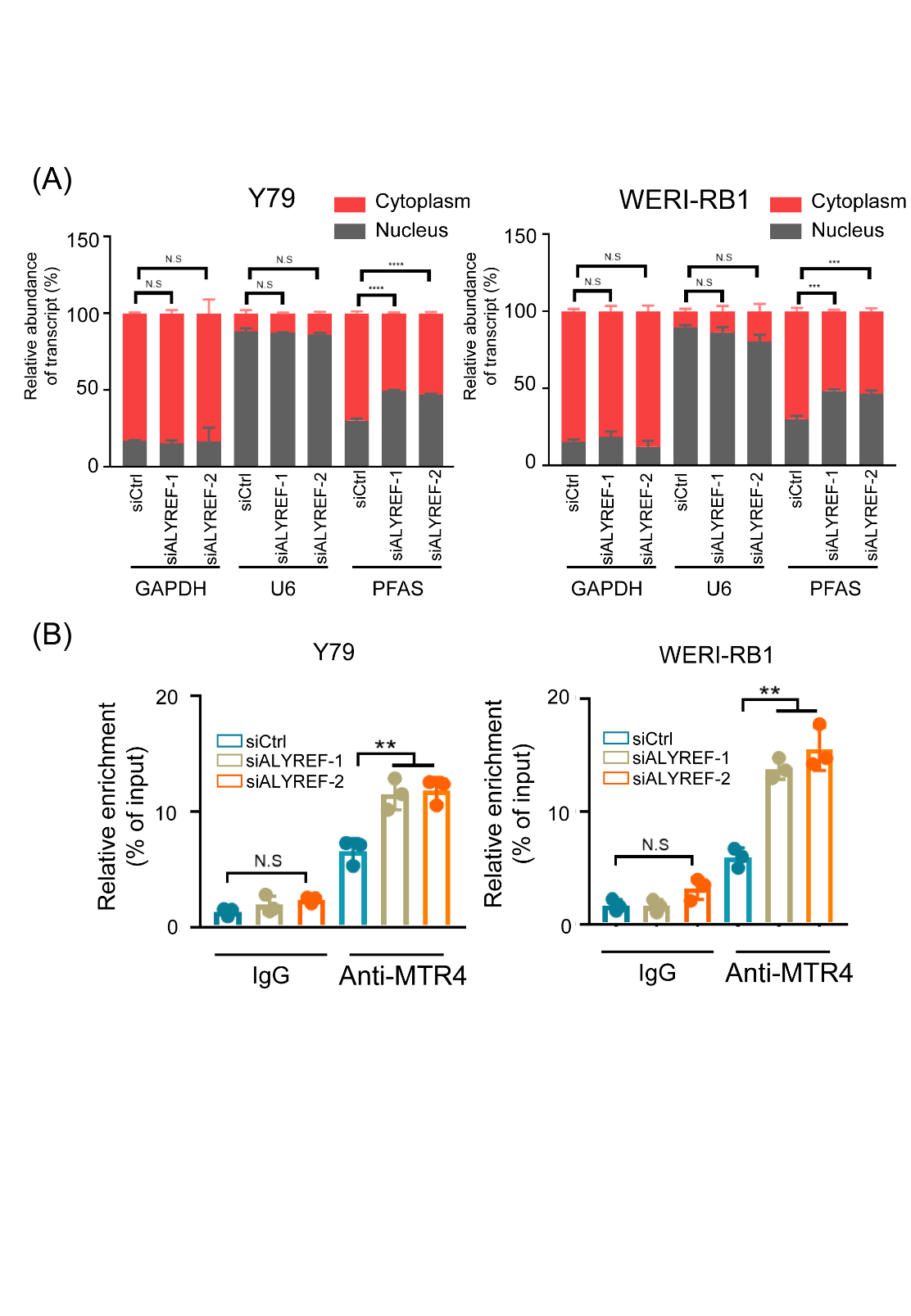


**FIGURE S13. ALYREF regulates PFAS RNA shuttling.**

(A) The proportion of PFAS RNA in nucleus and cytoplasm upon ALYREF knockdown. The data are presented as the mean ± SD of experimental triplicates. Significance was determined by an unpaired two-tailed Student’s t test. ***P< 0.001, ****P< 0.0001.N.S indicates no significance.

(B) RNA‐IP analysis showing the association of hMTR4 with PFAS is enhanced upon ALYREF knockdown. The data are presented as the mean ± SD of experimental triplicates. Significance was determined by an unpaired two-tailed Student’s t test. **P< 0.01, N.S indicates no significance.

**
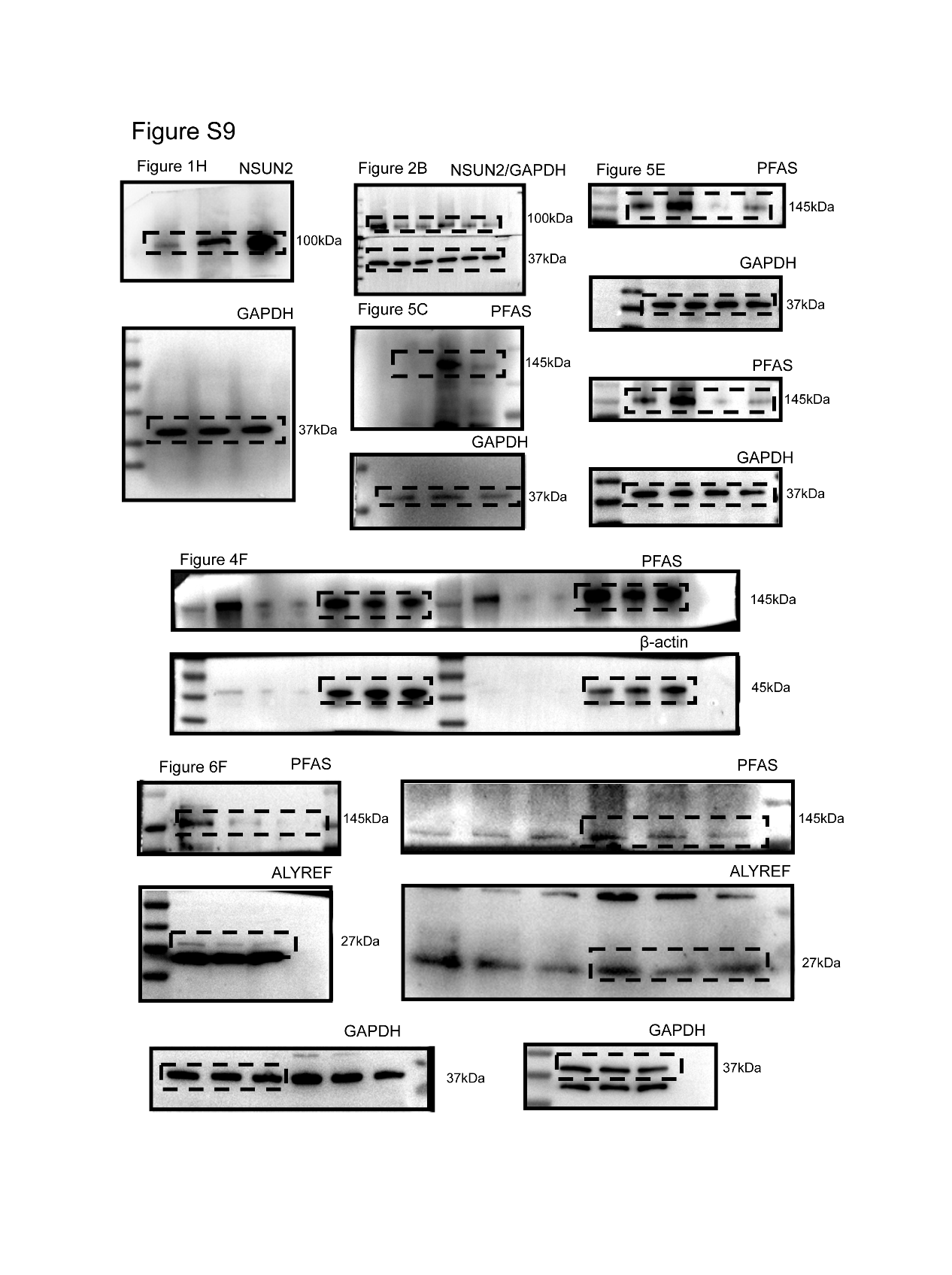
**

**FIGURE S14. Unprocessed western blot.**


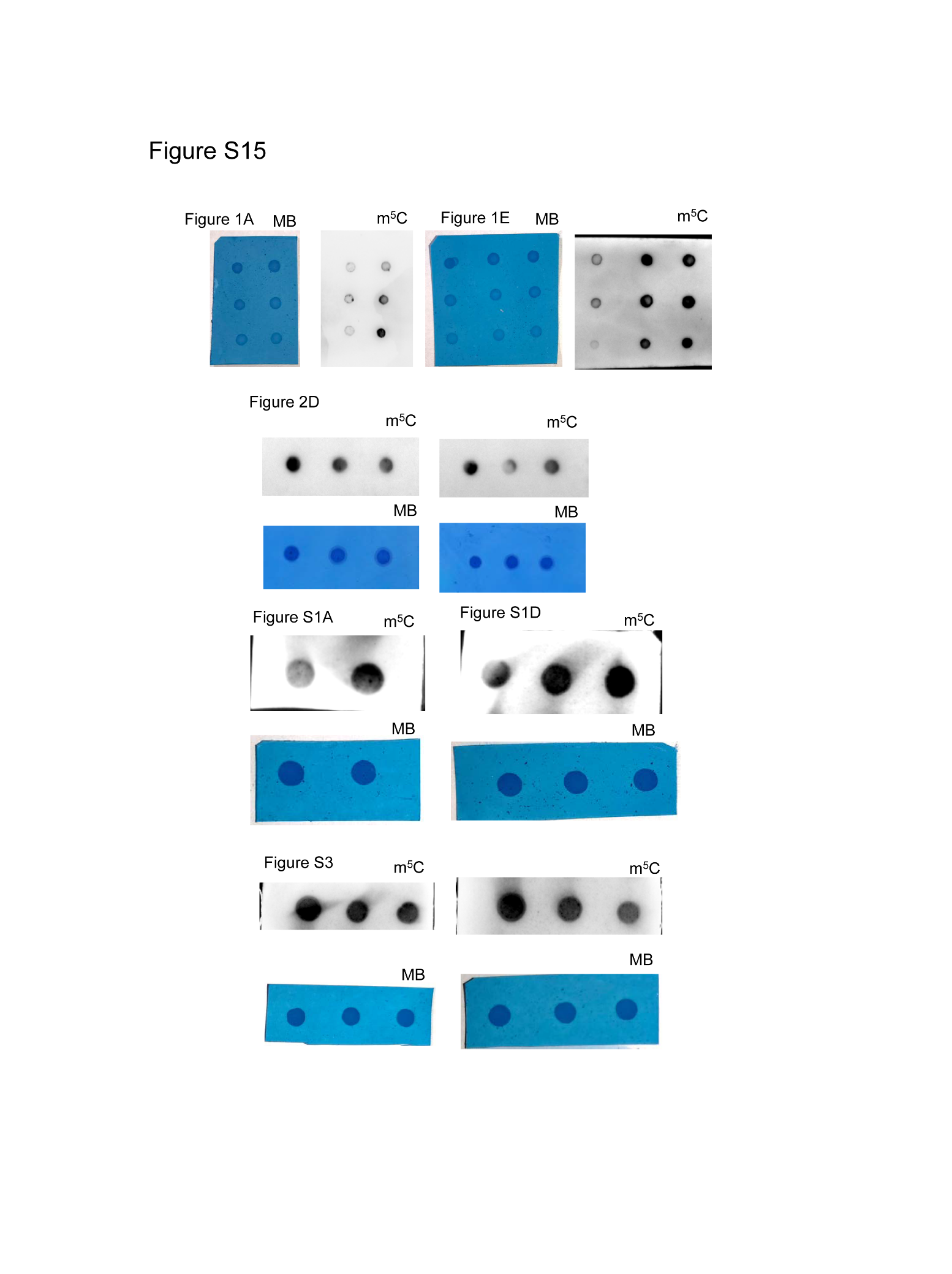


**FIGURE S15. Unprocessed dot blot images.**

**Table S1. The clinical characteristics of retinoblastoma patient cohorts**

| \| Features \| Retinoblastoma \| Adjacent normal retina tissue \| \| --- \| --- \| --- \| \| Numbers \| 3 \| 3 \| \| Sex \|  \|  \| \| Male \| 1 \| 1 \| \| Female \| 2 \| 2 \| \| Age \| 4.0±2.0 \| 3.5±1.5 \| \| Lateral \|  \|  \| \| Left \| 1 \| 2 \| \| Right \| 2 \| 1 \| \| Stage (IIRC) \|  \|  \| \| Group A \| 0 \| /  /  /  /  / \| \| Group B \| 0 \| \| Group C \| 0 \| \| Group D \| 0 \| \| Group E \| 3 \| |
| --- | --- | --- | --- | --- | --- | --- | --- | --- | --- | --- | --- | --- | --- | --- | --- | --- | --- | --- | --- | --- | --- | --- | --- | --- | --- | --- | --- | --- | --- | --- | --- | --- | --- | --- | --- | --- | --- | --- | --- | --- | --- |

|  |
| --- |

**Table S2. Primers, shRNA and siRNA used in the experiment**

| Gene | Forwards (5′-3′) | Reverse (5′-3′) |
| --- | --- | --- |
| NSUN2  PFAS  ALYREF  β-actin  GAPDH  U6 | GCAAGCTCCAAAGCACCTTC  GTGCATCTTCCACGAACCTAA  CCATGGCCGACAAAATGGAC  GAGCACAGAGCCTCGCCTTT  GGTCGGAGTCAACGGATTT  AAAGCAAATCATCGGACGACC | ATGTGGACGGCAGGAAAGAG  GTAAGGGGCAACCAAACAGC  CGGGAAGTTGTTTTGGCCTG  TCATCATCCATGGTGAGCTGG  CCAGCATCGCCCCACTTGA  GTACAACACATTGTTTCCTCGGA |

| shRNA | Sequence (5′-3′) |
| --- | --- |
| shNSUN2-1  shNSUN2-2 | CGAATGATGTGGACAACAA  TGAGAAGATGAAGGTTATTAA |
| siRNA | Sequence (5′-3′) |
| siALYREF-1  siALYREF-2  siPFAS-1  siPFAS-2 | GGAAACTGCTGGTGTCCAATC  CGTGGAGACAGGTGGGAAACT  GGAGGAAACTGCAAGGGAAAC  GGTCTGGCTTTAGACTCTTGG |

**Table S3. Proteins altered in retinoblastoma upon NSUN2 knockdown**

| **Accession** | **Gene** | ***P*-value** | **Fold change** |
| --- | --- | --- | --- |
| \| P06733 \| \| --- \| \| P04075 \| \| Q12906 \| \| P60174 \| \| P46821 \| \| P06744 \| \| P29401 \| \| O43707 \| \| P31948 \| \| P00338 \| \| P62258 \| \| P15311 \| \| P09972 \| \| P63104 \| \| Q99497 \| \| P16949 \| \| P52209 \| \| O15067 \| \| P07195 \| \| P50395 \| \| P15531 \| \| P29373 \| \| P50502 \| \| P38159 \| \| P17174 \| \| P22392 \| \| P40925 \| \| Q8NC51 \| \| P46926 \| \| Q9BZZ5 \| \| P31150 \| \| Q14103 \| \| Q9Y266 \| \| Q8N8S7 \| \| Q14978 \| \| P62987 \| \| P10599 \| \| Q9UK76 \| \| P39019 \| \| Q86V81 \| \| P30046 \| \| P09012 \| \| P61088 \| \| P51858 \| \| Q08J23 \| \| P30086 \| \| P23588 \| \| Q13765 \| \| P82979 \| \| P28070 \| \| P61970 \| \| P00441 \| \| P13798 \| \| Q14019 \| \| P62750 \| \| O14979 \| \| P26583 \| \| P20290 \| \| Q15819 \| \| Q99436 \| \| Q13404 \| \| P14174 \| \| P62857 \| \| P35637 \| \| Q16851 \| \| P49006 \| \| P53999 \| \| O14737 \| \| O15347 \| \| P28074 \| \| Q9Y3Y2 \| \| P61956 \| \| P67809 \| \| P63279 \| \| P48637 \| \| P37108 \| \| P16989 \| \| P61353 \| \| Q01130 \| \| P47914 \| \| Q00688 \| \| P08397 \| \| Q15427 \| \| Q15843 \| \| O75347 \| \| Q9P013 \| \| Q8IYB3 \| \| Q14919 \| \| P60983 \| \| P17096 \| \| P62942 \| \| Q13442 \| \| Q01844 \| \| O00479 \| \| Q9Y3E1 \| \| P63220 \| \| Q9BQ61 \| \| Q14247 \| \| Q01658 \| \| P52926 \| \| Q9BRA2 \| \| O43670 \| \| P62861 \| \| O60925 \| \| P84090 \| \| P20962 \| \| O75937 \| \| P06132 \| \| P63313 \| \| Q9H1E3 \| \| P09234 \| \| P62318 \| \| Q9Y2S6 \| \| P62891 \| \| Q96C90 \| \| O15514 \| \| P46597 \| \| Q9UPT8 \| \| O00193 \| \| Q9UL46 \| \| Q9H9S4 \| \| Q96C19 \| \| P05114 \| \| O43583 \| \| Q9GZQ8 \| \| Q9BTL3 \| \| Q9BRF8 \| \| P61927 \| \| O15116 \| \| Q9H299 \| \| P17655 \| \| Q9NZB8 \| \| Q08623 \| \| P02751 \| \| Q14697 \| \| P14314 \| \| Q9H4B7 \| \| P00734 \| \| Q9NZN3 \| \| Q6PI48 \| \| P69905 \| \| P53007 \| \| P06396 \| \| P30040 \| \| P12259 \| \| P01024 \| \| O00299 \| \| P07996 \| \| P04114 \| \| Q13418 \| \| P04179 \| \| P01031 \| \| P30613 \| \| P19823 \| \| P48426 \| \| P02649 \| \| Q99829 \| \| P02647 \| \| Q969S9 \| \| Q9BRR6 \| \| P35443 \| \| P01008 \| \| Q16772 \| \| P02042 \| \| Q99956 \| \| P01023 \| \| P69891 \| \| P02100 \| \| P10643 \| \| Q86UX7 \| \| P04004 \| \| P19525 \| \| O95248 \| \| P23142 \| \| O60942 \| \| P55212 \| \| O95445 \| \| P48059 \| \| P06737 \| \| P16152 \| \| Q9HBI1 \| \| O00506 \| \| P02675 \| \| P02774 \| \| Q6NUQ4 \| \| P22352 \| \| Q15063 \| \| P00488 \| \| P54709 \| \| P0C0L4 \| \| Q14520 \| \| Q13126 \| \| Q9H8M2 \| \| P20742 \| \| Q01970 \| \| P07358 \| \| P08567 \| \| Q15493 \| \| O14556 \| \| Q16401 \| \| Q8WVX9 \| \| P48740 \| \| Q2TAL8 \| \| Q93088 \| \| P02748 \| \| Q8TD19 \| \| Q01484 \| \| Q8NHP8 \| \| Q15555 \| \| Q02809 \| \| P02671 \| \| Q9NZ53 \| \| Q9HAD4 \| \| Q9Y240 \| \| Q56VL3 \| \| Q13472 \| \| Q6P1K2 \| \| Q9H1E5 \| \| Q86UY6 \| \| Q9H7B4 \| \| Q9BX79 \| \| O60218 \| \| P21810 \| \| Q9BWP8 \| \| P55058 \| \| P07093 \| \| P05546 \| \| Q6ZXV5 \| \| P30291 \| \| O60879 \| \| Q9Y6N6 \| \| P0C221 \| \| Q9NZR1 \| \| Q7RTV2 \| \| P00450 \| \| Q9HBL8 \| \| Q76LX8 \| \| O43294 \| \| Q05397 \| \| Q12899 \| \| Q14149 \| \| Q5TEZ5 \| \| Q8WXE9 \| \| Q8WYQ5 \| \| Q9NZP5 \| \| Q6ZN66 \| \| P00747 \| \| Q14624 \| \| P13671 \| \| O75891 \| \| Q9C000 \| \| Q7Z5H4 \| \| P48775 \| \| P34913 \| \| Q8NBM8 \| \| Q86YA3 \| \| Q03001 \| \| O75794 \| \| Q6XD76 \| \| Q6ZYL4 \| \| P51668 \| \| Q96DG6 \| \| P49888 \| \| Q9P2D0 \| \| Q9Y239 \| \| Q9NXJ5 \| \| Q6DN14 \| \| P52895 \| \| Q13227 \| \| Q5H9R4 \| \| P09467 \| \| Q6ZS92 \| \| Q9BV23 \| \| Q96MI9 \| \| Q5H9T9 \| \| Q8IXQ6 \| \| Q8NF67 \| \| Q96MP8 \| \| O95365 \| \| Q8NBH2 \| \| Q9UJP4 \| | \| ENO1 \| \| --- \| \| ALDOA \| \| ILF3 \| \| TPI1 \| \| MAP1B \| \| GPI \| \| TKT \| \| ACTN4 \| \| STIP1 \| \| LDHA \| \| YWHAE \| \| EZR \| \| ALDOC \| \| YWHAZ \| \| PARK7 \| \| STMN1 \| \| PGD \| \| PFAS \| \| LDHB \| \| GDI2 \| \| NME1 \| \| CRABP2 \| \| ST13 \| \| RBMX \| \| GOT1 \| \| NME2 \| \| MDH1 \| \| SERBP1 \| \| GNPDA1 \| \| API5 \| \| GDI1 \| \| HNRNPD \| \| NUDC \| \| ENAH \| \| NOLC1 \| \| UBA52 \| \| TXN \| \| JPT1 \| \| RPS19 \| \| ALYREF \| \| DDT \| \| SNRPA \| \| UBE2N \| \| HDGF \| \| NSUN2 \| \| PEBP1 \| \| EIF4B \| \| NACA \| \| SARNP \| \| PSMB4 \| \| NUTF2 \| \| SOD1 \| \| APEH \| \| COTL1 \| \| RPL23A \| \| HNRNPDL \| \| HMGB2 \| \| BTF3 \| \| UBE2V2 \| \| PSMB7 \| \| UBE2V1 \| \| MIF \| \| RPS28 \| \| FUS \| \| UGP2 \| \| MARCKSL1 \| \| SUB1 \| \| PDCD5 \| \| HMGB3 \| \| PSMB5 \| \| CHTOP \| \| SUMO2 \| \| YBX1 \| \| UBE2I \| \| GSS \| \| SRP14 \| \| YBX3 \| \| RPL27 \| \| SRSF2 \| \| RPL29 \| \| FKBP3 \| \| HMBS \| \| SF3B4 \| \| NEDD8 \| \| TBCA \| \| CWC15 \| \| SRRM1 \| \| DRAP1 \| \| GMFB \| \| HMGA1 \| \| FKBP1A \| \| PDAP1 \| \| EWSR1 \| \| HMGN4 \| \| HDGFL3 \| \| RPS21 \| \| TRIR \| \| CTTN \| \| DR1 \| \| HMGA2 \| \| TXNDC17 \| \| ZNF207 \| \| FAU \| \| PFDN1 \| \| ERH \| \| PTMS \| \| DNAJC8 \| \| UROD \| \| TMSB10 \| \| NUCKS1 \| \| SNRPC \| \| SNRPD3 \| \| TMA7 \| \| RPL39 \| \| PPP1R14B \| \| POLR2D \| \| ASMT \| \| ZC3H4 \| \| SMAP \| \| PSME2 \| \| CAB39L \| \| EFHD2 \| \| HMGN1 \| \| DENR \| \| MAP1LC3B \| \| RAMAC \| \| CPPED1 \| \| RPL37 \| \| LSM1 \| \| SH3BGRL3 \| \| CAPN2 \| \| MOCS1 \| \| PUDP \| \| FN1 \| \| GANAB \| \| PRKCSH \| \| TUBB1 \| \| F2 \| \| EHD3 \| \| DARS2 \| \| HBA1 \| \| SLC25A1 \| \| GSN \| \| ERP29 \| \| F5 \| \| C3 \| \| CLIC1 \| \| THBS1 \| \| APOB \| \| ILK \| \| SOD2 \| \| C5 \| \| PKLR \| \| ITIH2 \| \| PIP4K2A \| \| APOE \| \| CPNE1 \| \| APOA1 \| \| GFM2 \| \| ADPGK \| \| THBS4 \| \| SERPINC1 \| \| GSTA3 \| \| HBD \| \| DUSP9 \| \| A2M \| \| HBG1 \| \| HBE1 \| \| C7 \| \| FERMT3 \| \| VTN \| \| EIF2AK2 \| \| SBF1 \| \| FBLN1 \| \| RNGTT \| \| CASP6 \| \| APOM \| \| LIMS1 \| \| PYGL \| \| CBR1 \| \| PARVB \| \| STK25 \| \| FGB \| \| GC \| \| TMEM214 \| \| GPX3 \| \| POSTN \| \| F13A1 \| \| ATP1B3 \| \| C4A \| \| HABP2 \| \| MTAP \| \| BRD9 \| \| PZP \| \| PLCB3 \| \| C8B \| \| PLEK \| \| RGN \| \| GAPDHS \| \| PSMD5 \| \| FAR1 \| \| MASP1 \| \| QRICH1 \| \| BHMT \| \| C9 \| \| NEK9 \| \| ANK2 \| \| PLBD2 \| \| MAPRE2 \| \| PLOD1 \| \| FGA \| \| PODXL2 \| \| WDR41 \| \| CLEC11A \| \| OCIAD2 \| \| TOP3A \| \| PMF1 \| \| TMX4 \| \| NAA40 \| \| SMYD3 \| \| STRA6 \| \| AKR1B10 \| \| BGN \| \| COLEC11 \| \| PLTP \| \| SERPINE2 \| \| SERPIND1 \| \| TMTC3 \| \| WEE1 \| \| DIAPH2 \| \| LAMC3 \| \| CCDC175 \| \| TMOD2 \| \| GSTA5 \| \| CP \| \| NMRAL1 \| \| ADAMTS13 \| \| TGFB1I1 \| \| PTK2 \| \| TRIM26 \| \| MORC3 \| \| C6orf163 \| \| STON2 \| \| DGCR8 \| \| OR5AC2 \| \| GBP6 \| \| PLG \| \| ITIH4 \| \| C6 \| \| ALDH1L1 \| \| NLRP1 \| \| VN1R5 \| \| TDO2 \| \| EPHX2 \| \| PCYOX1L \| \| ZGRF1 \| \| DST \| \| CDC123 \| \| ASCL4 \| \| GTF2H5 \| \| UBE2D1 \| \| CMBL \| \| SULT1E1 \| \| IBTK \| \| NOD1 \| \| PGPEP1 \| \| MCTP1 \| \| AKR1C2 \| \| GPS2 \| \| ARMCX4 \| \| FBP1 \| \| /  ABHD6 \| \| AGBL1 \| \| FSCB \| \| PARP9 \| \| ANKRD20A12P \| \| KCTD7 \| \| ZBTB7A \| \| KY \| \| KLHL21 \| | \| 0.016191 \| \| --- \| \| 0.005156 \| \| 0.001961 \| \| 0.01551 \| \| 0.00157 \| \| 0.003872 \| \| 0.004524 \| \| 0.001446 \| \| 0.004271 \| \| 0.006246 \| \| 0.010865 \| \| 0.004639 \| \| 0.000895 \| \| 0.007806 \| \| 0.004656 \| \| 0.012541 \| \| 0.007323 \| \| 0.003558 \| \| 0.014033 \| \| 0.00615 \| \| 0.009507 \| \| 0.00743 \| \| 0.005046 \| \| 0.001664 \| \| 0.00278 \| \| 0.021034 \| \| 0.00558 \| \| 0.001969 \| \| 0.001017 \| \| 0.00129 \| \| 0.005154 \| \| 0.013968 \| \| 0.002213 \| \| 0.00273 \| \| 0.005572 \| \| 0.038976 \| \| 0.012284 \| \| 0.003103 \| \| 0.004504 \| \| 0.001805 \| \| 0.009083 \| \| 0.001541 \| \| 0.001821 \| \| 0.002357 \| \| 0.001392 \| \| 0.00726 \| \| 0.00496 \| \| 0.002293 \| \| 0.001267 \| \| 0.004664 \| \| 0.014675 \| \| 0.004636 \| \| 0.002078 \| \| 0.004865 \| \| 0.007745 \| \| 0.002988 \| \| 0.00891 \| \| 0.002307 \| \| 0.012795 \| \| 0.004747 \| \| 0.004327 \| \| 0.008705 \| \| 0.035333 \| \| 0.005767 \| \| 0.001922 \| \| 0.019751 \| \| 0.001378 \| \| 0.028122 \| \| 0.020787 \| \| 0.010883 \| \| 0.002903 \| \| 0.003941 \| \| 0.009274 \| \| 0.017035 \| \| 0.000144 \| \| 0.003336 \| \| 0.004675 \| \| 0.021488 \| \| 0.008607 \| \| 0.005272 \| \| 0.003595 \| \| 0.00021 \| \| 0.009443 \| \| 0.003313 \| \| 0.008324 \| \| 0.005551 \| \| 0.029544 \| \| 0.001837 \| \| 0.000137 \| \| 0.001874 \| \| 0.000873 \| \| 0.000303 \| \| 0.008583 \| \| 0.006933 \| \| 0.003308 \| \| 0.005072 \| \| 0.008451 \| \| 0.018733 \| \| 0.005351 \| \| 0.020934 \| \| 0.000972 \| \| 0.023362 \| \| 0.004437 \| \| 0.0002 \| \| 0.007173 \| \| 0.006946 \| \| 0.003704 \| \| 0.015322 \| \| 0.000894 \| \| 0.00104 \| \| 0.003791 \| \| 0.009051 \| \| 0.001752 \| \| 0.017528 \| \| 0.020675 \| \| 0.001899 \| \| 0.030189 \| \| 0.001034 \| \| 0.003964 \| \| 0.016608 \| \| 0.003292 \| \| 0.009715 \| \| 0.002323 \| \| 0.0104 \| \| 0.007007 \| \| 0.011786 \| \| 0.011166 \| \| 0.012371 \| \| 0.002024 \| \| 0.007717 \| \| 0.019187 \| \| 0.00906 \| \| 0.002276 \| \| 0.036332 \| \| 0.001247 \| \| 0.007989 \| \| 0.004804 \| \| 0.000362 \| \| 0.019024 \| \| 0.026189 \| \| 0.010286 \| \| 0.002413 \| \| 0.00459 \| \| 0.014531 \| \| 0.013002 \| \| 0.008037 \| \| 0.004934 \| \| 0.006268 \| \| 0.038496 \| \| 0.005813 \| \| 0.044807 \| \| 0.019515 \| \| 0.016074 \| \| 0.014595 \| \| 0.010431 \| \| 0.043183 \| \| 0.005648 \| \| 0.011858 \| \| 0.022146 \| \| 0.021917 \| \| 0.011781 \| \| 0.024491 \| \| 0.009883 \| \| 0.000977 \| \| 0.001891 \| \| 0.009935 \| \| 0.001877 \| \| 0.011663 \| \| 0.005112 \| \| 0.006274 \| \| 0.004511 \| \| 0.006758 \| \| 0.019719 \| \| 0.001349 \| \| 0.02312 \| \| 0.006274 \| \| 0.00276 \| \| 0.001511 \| \| 0.004702 \| \| 0.014007 \| \| 0.00557 \| \| 0.001148 \| \| 0.016732 \| \| 0.004978 \| \| 0.001931 \| \| 0.005364 \| \| 0.003907 \| \| 0.02073 \| \| 0.005692 \| \| 0.02959 \| \| 0.003607 \| \| 0.016001 \| \| 0.006449 \| \| 0.025157 \| \| 0.034088 \| \| 0.012535 \| \| 0.006289 \| \| 0.00029 \| \| 0.014968 \| \| 0.020894 \| \| 0.001962 \| \| 0.000188 \| \| 0.03638 \| \| 0.036425 \| \| 0.042655 \| \| 0.018127 \| \| 0.025412 \| \| 0.012379 \| \| 0.001403 \| \| 0.021262 \| \| 0.009781 \| \| 0.010436 \| \| 0.014923 \| \| 0.000826 \| \| 0.000662 \| \| 0.016103 \| \| 0.005405 \| \| 0.006531 \| \| 0.023829 \| \| 0.001939 \| \| 0.015004 \| \| 0.01987 \| \| 0.014987 \| \| 0.003273 \| \| 0.020925 \| \| 0.027711 \| \| 0.00377 \| \| 0.002912 \| \| 0.008455 \| \| 0.011266 \| \| 0.026831 \| \| 0.022023 \| \| 0.019141 \| \| 0.01828 \| \| 0.006982 \| \| 0.00726 \| \| 0.020689 \| \| 0.008901 \| \| 0.035308 \| \| 0.035055 \| \| 0.005765 \| \| 0.04585 \| \| 0.022076 \| \| 0.018992 \| \| 0.031502 \| \| 0.014178 \| \| 0.002679 \| \| 0.025009 \| \| 0.009247 \| \| 0.021048 \| \| 0.015625 \| \| 0.014445 \| \| 0.010833 \| \| 0.008875 \| \| 0.020954 \| \| 0.037463 \| \| 0.019159 \| \| 0.012535 \| \| 0.016205 \| \| 0.0318 \| \| 0.01439 \| \| 0.005622 \| \| 0.000458 \| \| 0.005305 \| \| 0.028814 \| \| 0.015784 \| \| 0.030773 \| \| 0.018847 \| \| 0.016542 \| \| 0.04719 \| \| 0.011207 \| \| 0.023442 \| \| 0.024923 \| \| 0.017658 \| \| 0.031546 \| \| 0.025369 \| \| 0.025877 \| \| 0.038678 \| \| 0.014687 \| \| 0.008623 \| \| 0.017988 \| | \| 0.645825 \| \| --- \| \| 0.571485 \| \| 0.611604 \| \| 0.518027 \| \| 0.634655 \| \| 0.602806 \| \| 0.610954 \| \| 0.653576 \| \| 0.649485 \| \| 0.489758 \| \| 0.629328 \| \| 0.631579 \| \| 0.580174 \| \| 0.612006 \| \| 0.660025 \| \| 0.542615 \| \| 0.646091 \| \| 0.665279 \| \| 0.519559 \| \| 0.593862 \| \| 0.514578 \| \| 0.544998 \| \| 0.578532 \| \| 0.640689 \| \| 0.647858 \| \| 0.533154 \| \| 0.608363 \| \| 0.573564 \| \| 0.599121 \| \| 0.641626 \| \| 0.651528 \| \| 0.540239 \| \| 0.601922 \| \| 0.642036 \| \| 0.581257 \| \| 0.651528 \| \| 0.618123 \| \| 0.625356 \| \| 0.570699 \| \| 0.553786 \| \| 0.629992 \| \| 0.563111 \| \| 0.614609 \| \| 0.608119 \| \| 0.468062 \| \| 0.557632 \| \| 0.552795 \| \| 0.588959 \| \| 0.646502 \| \| 0.645413 \| \| 0.635063 \| \| 0.596806 \| \| 0.654944 \| \| 0.580632 \| \| 0.632395 \| \| 0.582509 \| \| 0.648125 \| \| 0.610709 \| \| 0.593625 \| \| 0.646091 \| \| 0.617469 \| \| 0.592357 \| \| 0.529052 \| \| 0.586043 \| \| 0.652893 \| \| 0.534714 \| \| 0.643385 \| \| 0.646768 \| \| 0.540031 \| \| 0.624949 \| \| 0.635323 \| \| 0.57876 \| \| 0.596806 \| \| 0.614205 \| \| 0.632395 \| \| 0.657415 \| \| 0.619433 \| \| 0.646091 \| \| 0.566784 \| \| 0.642975 \| \| 0.583531 \| \| 0.652893 \| \| 0.64271 \| \| 0.665279 \| \| 0.512665 \| \| 0.602564 \| \| 0.607473 \| \| 0.652893 \| \| 0.652479 \| \| 0.589428 \| \| 0.500938 \| \| 0.594261 \| \| 0.56189 \| \| 0.601922 \| \| 0.614857 \| \| 0.601922 \| \| 0.563722 \| \| 0.66182 \| \| 0.629072 \| \| 0.498127 \| \| 0.660025 \| \| 0.63707 \| \| 0.526135 \| \| 0.645002 \| \| 0.528468 \| \| 0.48368 \| \| 0.652893 \| \| 0.666667 \| \| 0.440043 \| \| 0.540832 \| \| 0.569859 \| \| 0.640952 \| \| 0.591325 \| \| 0.624695 \| \| 0.602323 \| \| 0.612253 \| \| 0.635732 \| \| 0.624695 \| \| 0.593862 \| \| 0.666667 \| \| 0.641626 \| \| 0.634395 \| \| 0.509434 \| \| 0.645413 \| \| 0.61006 \| \| 0.661545 \| \| 0.62206 \| \| 0.565558 \| \| 0.587302 \| \| 0.575197 \| \| 0.609658 \| \| 0.529052 \| \| 0.404988 \| \| 2.183917 \| \| 1.676254 \| \| 1.610313 \| \| 2.212851 \| \| 2.141398 \| \| 1.5 \| \| 1.538706 \| \| 2.169572 \| \| 1.506892 \| \| 1.590674 \| \| 1.501563 \| \| 1.686367 \| \| 1.984328 \| \| 1.724114 \| \| 1.677376 \| \| 1.86123 \| \| 1.654944 \| \| 1.793296 \| \| 1.728513 \| \| 1.982849 \| \| 2.177125 \| \| 1.58565 \| \| 1.636783 \| \| 1.554278 \| \| 2.202562 \| \| 1.55364 \| \| 1.516352 \| \| 2.301155 \| \| 2.265306 \| \| 2.395586 \| \| 1.886003 \| \| 1.522068 \| \| 1.846975 \| \| 1.952768 \| \| 1.97619 \| \| 1.73224 \| \| 1.881844 \| \| 2.103957 \| \| 1.520479 \| \| 1.820169 \| \| 1.793296 \| \| 1.598052 \| \| 1.553287 \| \| 2.327787 \| \| 1.626395 \| \| 1.659574 \| \| 1.632237 \| \| 1.772003 \| \| 1.708192 \| \| 2.152088 \| \| 1.663782 \| \| 1.542276 \| \| 2.081664 \| \| 2.458081 \| \| 1.700878 \| \| 1.515723 \| \| 1.642008 \| \| 2.189793 \| \| 1.684564 \| \| 2.581916 \| \| 1.527813 \| \| 1.609263 \| \| 1.565747 \| \| 1.616089 \| \| 1.787456 \| \| 2.037206 \| \| 1.557545 \| \| 1.663116 \| \| 2.350084 \| \| 1.676254 \| \| 1.666667 \| \| 1.955654 \| \| 1.568036 \| \| 1.695418 \| \| 1.985821 \| \| 1.631579 \| \| 1.590026 \| \| 2.392706 \| \| 1.698381 \| \| 1.627464 \| \| 2.246753 \| \| 2.051106 \| \| 1.508783 \| \| 1.523659 \| \| 1.542912 \| \| 1.552648 \| \| 1.664224 \| \| 1.542912 \| \| 2.069839 \| \| 1.987304 \| \| 2.366162 \| \| 1.793994 \| \| 1.617147 \| \| 1.615033 \| \| 1.526848 \| \| 1.621232 \| \| 1.60143 \| \| 1.74417 \| \| 2.014318 \| \| 2.149606 \| \| 2.009029 \| \| 2.334167 \| \| 1.553287 \| \| 2.009782 \| \| 1.586675 \| \| 1.659574 \| \| 1.725477 \| \| 1.863278 \| \| 1.526216 \| \| 2.270646 \| \| 1.533249 \| \| 1.826855 \| \| 2.095975 \| \| 2.281378 \| \| 2.139717 \| \| 1.857857 \| \| 1.728513 \| \| 2.780718 \| \| 1.904866 \| \| 1.975446 \| \| 1.700203 \| \| 1.590674 \| \| 1.58565 \| \| 1.680965 \| \| 1.602472 \| \| 1.501563 \| \| 1.779708 \| \| 1.807018 \| \| 1.855103 \| \| 2.218825 \| \| 1.725477 \| \| 1.642668 \| \| 1.75672 \| \| 1.647253 \| \| 1.682763 \| \| 1.597403 \| \| 1.805049 \| \| 2.076923 \| \| 1.81294 \| \| 1.57566 \| \| 1.985821 \| \| 1.818887 \| \| 1.749828 \| \| 1.746566 \| \| 1.762431 \| \| 1.940441 \| \| 1.857143 \| \| 1.901378 \| |
